# Small Molecule Accurate Recognition Technology (SMART) to Enhance Natural Products Research

**DOI:** 10.1038/s41598-017-13923-x

**Published:** 2017-10-27

**Authors:** Chen Zhang, Yerlan Idelbayev, Nicholas Roberts, Yiwen Tao, Yashwanth Nannapaneni, Brendan M. Duggan, Jie Min, Eugene C. Lin, Erik C. Gerwick, Garrison W. Cottrell, William H. Gerwick

**Affiliations:** 1 Department of Nanoengineering, University of California, San Diego, La Jolla, California, 92093 United States of America; 2 Department of Computer Science and Engineering, University of California, San Diego, La Jolla, California, 92093 United States of America; 3 Center for Marine Biotechnology and Biomedicine, Scripps Institution of Oceanography, La Jolla, California, 92037 United States of America; 4 School of Pharmaceutical Sciences, Guangzhou Medical University, Guangzhou, Guangdong, 511436 People’s Republic of China; 5 Skaggs School of Pharmacy and Pharmaceutical Sciences, University of California, San Diego, La Jolla, California, 92093 United States of America; 6 Department of Electrical and Computer Engineering, University of California, San Diego, La Jolla, California, 92093 United States of America; 7 Vanderbilt University Institute of Imaging Science, Vanderbilt University, Nashville, Tennessee 37235 United States of America; 8 Department of Radiology and Radiological Sciences, Vanderbilt University, Nashville, Tennessee 37235 United States of America; 9 Physikalisches Institut, Universität Göttingen, Friedrich-Hund-Platz 1, 37077 Göttingen, Germany

## Abstract

Various algorithms comparing 2D NMR spectra have been explored for their ability to dereplicate natural products as well as determine molecular structures. However, spectroscopic artefacts, solvent effects, and the interactive effect of functional group(s) on chemical shifts combine to hinder their effectiveness. Here, we leveraged Non-Uniform Sampling (NUS) 2D NMR techniques and deep Convolutional Neural Networks (CNNs) to create a tool, SMART, that can assist in natural products discovery efforts. First, an NUS heteronuclear single quantum coherence (HSQC) NMR pulse sequence was adapted to a state-of-the-art nuclear magnetic resonance (NMR) instrument, and data reconstruction methods were optimized, and second, a deep CNN with contrastive loss was trained on a database containing over 2,054 HSQC spectra as the training set. To demonstrate the utility of SMART, several newly isolated compounds were automatically located with their known analogues in the embedded clustering space, thereby streamlining the discovery pipeline for new natural products.

## Introduction

As a discipline, natural products research (NPR) enables and benefits numerous downstream research fields, such as chemical biology, chemical ecology, drug discovery and development, pharmacology and the total chemical synthesis of natural products (NPs). In this regard, approximately 70% of all approved drugs are NPs, their analogues, or a chemical modification of an existing NP^1^. In addition to these academic and societal benefits, NPR provides a powerful incentive for the conservation and sustainable use of biodiversity and biodiverse habitats^2^.

An important step in NPR is dereplication, the process of assessing the uniqueness of a new compound in relationship to all known ones. In most NPR, traditional compound dereplication practices have entailed the collection and analysis of nuclear magnetic resonance (NMR) spectra, including running 1D and 2D NMR spectroscopic experiments for the purposes of molecular framework construction, assembly, and relative stereochemistry determination. More recently, mass spectrometric approaches and mass spectrometry (MS)-based molecular networking^3^, in part stimulated by integration with DNA sequencing and genome mining^4,5^ have been integrated into NPR workflows. Nevertheless, conventional NMR practices are still indispensable to the characterization and dereplication of NPs. Unfortunately, 2D NMR experiments can be time consuming, especially when the sample is relatively scarce. Furthermore, 2D NMR-based structural assignments can sometimes take protracted periods of time to accomplish due to the inherent structural complexity of some NPs.

Along with relatively recent improvements in mass spectrometry, circular dichroism and infrared spectroscopy techniques, state-of-the-art cryoprobe NMR instruments have reduced the sample requirements for NPs discovery to just a few nanomoles^6^. Nevertheless, acquisition of NMR spectra may still require a relatively large number of time consuming scans before Fourier transformation of the data. Furthermore, conventional 2D NMR spectroscopy relies upon linear sampling of the frequency evolution in the indirect dimension (usually the ^13^C dimension). When generating high frequency resolution in the indirect dimension, extensive sampling is required and the experiments become very time consuming. Modification of conventional uniform sampling to non-uniform sampling (NUS)^7–13^ allows the number of experiments in the indirect dimension to be reduced, thereby reducing the overall time of the experiment. The NUS method is designed to reduce the number of data collection experiments while at the same time delivering an accurate estimation of the fully sampled spectrum.

To streamline compound dereplication or even structure determination, algorithms have been applied for 2D NMR spectra comparisons, such as the 2D NMR peak alignment algorithm^14,15^. However, these techniques are not powerful enough to accurately classify 2D NMR spectra into the correct NP family. This arises for several reasons, such as compound concentration, solvent effects, and the interactive effect of a single functional group alteration on ^1^H and ^13^C NMR chemical shifts, all of which combine to increase the difficulty for computer assisted 2D NMR data analysis. At the same time, artefacts are often introduced into NMR spectra, and this makes it difficult for existing pattern recognition or overlap methods to distinguish genuine peaks from artefacts. However, artificial intelligence technologies, such as deep learning^16,17^, have generated new approaches for meeting these challenges. Compared with conventional machine learning methods, which require the cumbersome process of selecting and creating features that might be suboptimal for a given task, deep learning allows creation of the most suitable set of features within the process of training, without any design or involvement by the investigator. Moreover, some deep learning methods work well even when the number of categories is very large and unknown during the training process. Thus, deep learning is an ideal method by which to analyse and categorize 2D NMR spectra of NPs. For NPs, there are an essentially unlimited number of categories for different compound families, with many being unknown at the present time. Additionally, it is quite common for each category to contain fewer than 50 different members; in the work of our laboratory with marine cyanobacterial NPs, this is the case for all of the molecular families we have encountered over the past 40 years, including the curacins^18–20^, apratoxins^21^, lyngbyabellins^22^ and majusculamides^23–25^.

Other approaches for automatic recognition of NMR spectra have appeared in the literature or private sector. The typical approach is to create grids over the data and then compute similarities based on how many points fall into the same grid cells^26^. This approach can miss peaks that are near one another that happen to fall in different grid cells, so an enhancement of this approach is to use multiple grid resolutions and offsets before computing the similarities^27^. Our convolutional network approach automatically does this by using overlapping convolutions combined with increasing-sized receptive fields through pooling the results from previous layers. However, our method of deciding similarity is learned by the network through nonlinear dimensionality reduction via training it to map together those compounds it recognizes as being from the same family, and to map different families to different locations in the underlying space.

Another method involves computer-aided structure elucidation (CASE, ACD/Labs) which is largely based on applying a least-squares regression (LSR) approach for comparing NMR chemical shifts; this tactic is not powerful enough to satisfactorily accommodate solvent effects, instrumental artefacts, or weak signal issues^14,15^. An early effort using machine learning applied to NMR spectra was reported in (Wolfram *et al*., 2006)^28^. They used Probabilistic Latent Semantic Indexing (PLSI), a method usually applied to text documents for information retrieval purposes. PLSI maps documents into a lower dimensional space using a probabilistic analogue to Singular Value Decomposition (SVD) applied to a document by word count matrix. To apply PLSI to compounds, the typical multi-scale and shifted grid cell approach was used, treating each grid cell as a “word” in the compound. This is essentially learning a linear mapping from the feature space to a reduced space, and thus is not as powerful as using a nonlinear deep network.

In our approach, heteronuclear single quantum correlation (HSQC)^29^ spectra are recorded using a 2D NMR pulse sequence that uses the large heteronuclear coupling between directly bonded nuclei within an organic molecule to correlate directly bonded atoms (*e.g*. ^1^H and ^13^C, with ^1^H being defined as the direct dimension and ^13^C the indirect dimension). The peaks of those correlated nuclei in the 2D HSQC spectra are generated by detecting coherences that connect states whose total *z*-angular momentum quantum numbers differ by one order (*i.e*. single-quantum coherences). In this regard, an HSQC spectrum is deemed as the ‘fingerprint’ or ‘face’ for a natural product molecule, and thus is highly discriminating. Specifically, within a 2D HSQC spectrum, signals in the direct dimension can be distinguished if they have shifts of 0.02 ppm or greater, and in the indirect dimension if they have shifts of 0.1 ppm or greater. Furthermore, most ^1^H chemical shifts occur between 0.5 and 9.5 ppm, whereas in the ^13^C dimension chemical shifts typically occur between 10 and 215 ppm, which gives rise to 922,500 distinguishable positions within a 2D HSQC spectrum. When summed over all protonated carbons in a molecule of 20 carbons with attached protons, the number of potential combinations is in the tens of millions, and is thus highly discriminatory. In addition, this technique avoids detection of double-quantum coherence, resulting in relatively few artefacts. In contrast, the commonly used heteronuclear multiple bond correlation (HMBC) experiment detects two and three bond correlations by selecting smaller multiple bond heteronuclear coupling constants (around 5–10 Hz for ^1^H-^13^C versus one bond of 125–170 Hz) for double-quantum and zero-quantum transfer. Therefore, while the HMBC experiment produces an even larger amount of theoretical information, it is prone to introducing artefacts and its complexity makes it more difficult to interpret. In addition, the HSQC when performed with NUS discussed above is a relatively quick and efficient experiment for data accumulation.

Here, we report the development of the Small Molecule Accurate Recognition Technology (SMART) prototype, a system that integrates the benefits of NUS NMR with advances in deep learning to enhance and improve the efficiency of NP dereplication. To create SMART, a database of training examples containing 2D HSQC spectra of 2,054 compounds was compiled. These examples were used to train a deep network that learns to map the spectra into a cluster space where similar compounds are near one another and dissimilar compounds are far apart. To perform this function, we use a deep convolutional neural network (CNN) employing a siamese architecture^30^ as described in the methods section. A siamese network amplifies the number of training examples by training on pairs of spectra that are labelled “same” or “different,” rather than training on individual examples. The network then learns features of the spectra that are relevant to their similarities and differences, and uses this to create the cluster space. The resulting mapping then generalizes to new compounds, placing them in the space near compounds with similar HSQC spectra. We evaluate SMART by holding back several known NPs from different families from the training set, and then show that SMART maps them into their proper location within the cluster space. We also present here the rapid identification of a newly isolated natural product compound family as a result of SMART’s ability to cluster similar compounds together. HSQC spectra were collected for several nonribosomal peptide synthetase (NRPSs)-derived NPs that had been isolated from two marine cyanobacteria. These novel spectra were accurately mapped by SMART into the ‘viequeamide’ subfamily of NPs.

## Results and Discussion

### The SMART prototype

**Figure 1:**
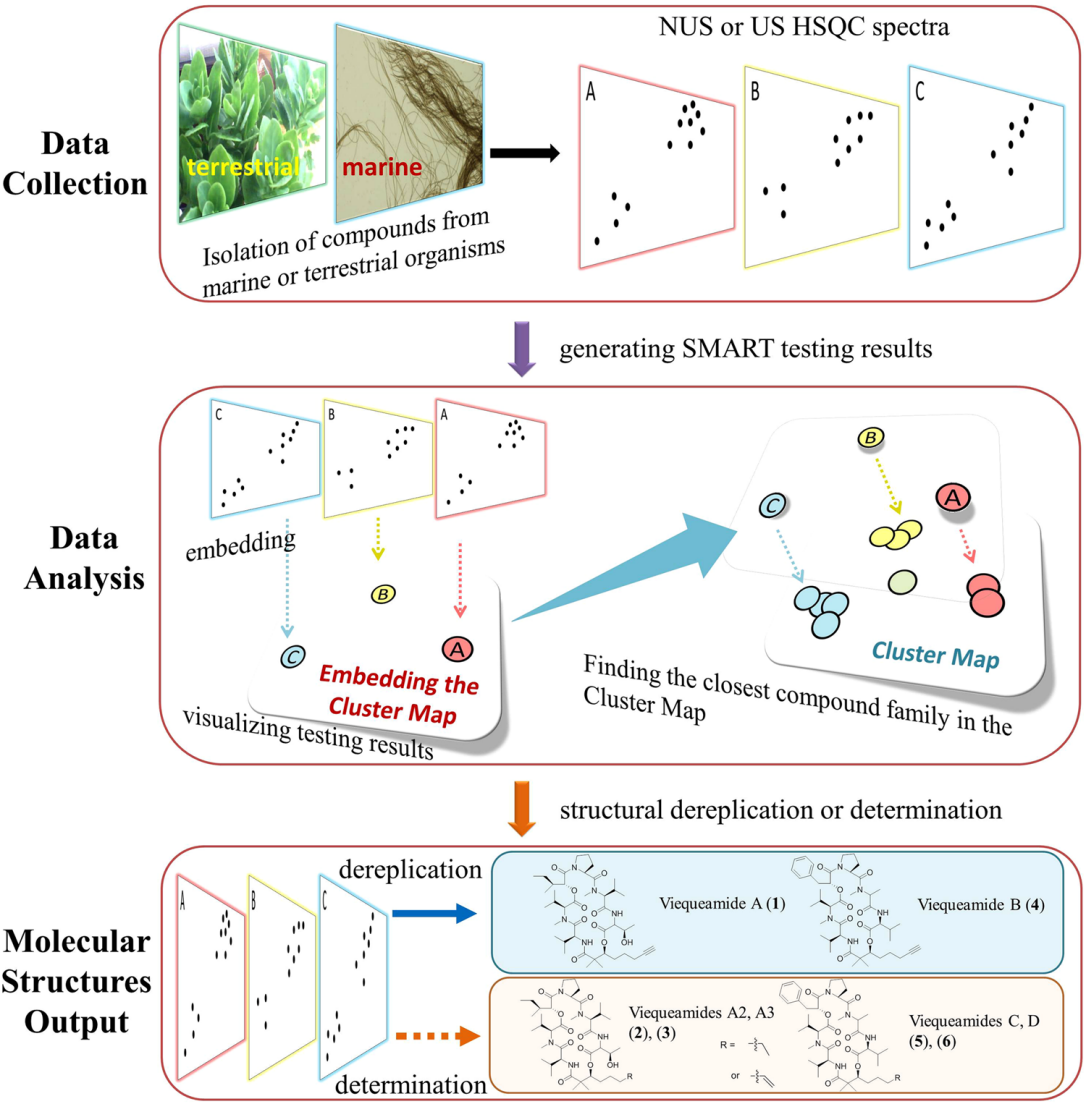
Workflow for the Small Molecule Accurate Recognition Technology (SMART). Experimental HSQC spectra of newly isolated pure natural product molecules collected using either NUS HSQC pulse sequences or conventional HSQC techniques, are automatically embedded by SMART into a cluster space near similar, previously-characterized compounds. The resultant embedding in the cluster map is visualized using the Bokeh visualization package^72^, where each node represents an HSQC spectrum processed by SMART. The node colours in a local area of the clustering map designate compounds from the same journal articles and thus of the same natural product family. This facile method allowed tracking of compounds into SMART, but is not of paramount significance in that some compounds reported in different publications display closer relationships in SMART and by structural comparison than to compounds within the same article. When available, the node labels are the compound names; otherwise, the labels are for the organism from which the compound derives. Node distance is proportional to relatedness, a quantification of molecular structural similarity. The 2D cluster map is created by performing Principal Component Analysis (PCA) of the 10D space outputs to reduce to 2D. Optionally, the top 5, 10 and 20 closest nodes in the 10D space are available in text format. The proof-of-concept experiments are illustrated: Dereplication (solid blue arrow) of viequeamides A (**1**) and B (**4**), and determination (dashed orange arrow) of viequeamides A2 (**2**), A3 (**3**), C (**5**) and D (**6**), isolated from 1) *Rivularia sp*., collected in Vieques, Puerto Rico and 2) *Moorea sp*., collected in American Samoa.

SMART is a user-friendly, AI-based dereplication and analysis tool that uses 2D NMR data to rapidly associate newly isolated NPs with their known analogues. SMART has been designed to mimic the normal path of experiential learning in that additional 2D NMR spectral inputs can be used to enrich its database and improve its performance. In short, SMART aims to become an experienced associate to natural products researchers as well as other classes of organic chemists. The SMART workflow consists of three steps, 1) 2D NMR data acquisition by NUS HSQC pulse sequence, 2) 2D NMR spectral analysis by deep CNN, resulting in an embedding of the spectra into a similarity space of NPs, and 3) molecular structure dereplication or determination by the user (Fig. 1). This process gives users rapid access to a well-organized map of structurally determined NPs, and helps ensure that SMART’s insights are chemically rational. In this regard, SMART capitalizes on the wealth of molecular fingerprints, namely 2D HSQC spectra, built over the past four decades^31,32^, and reciprocally, we anticipate that 2D HSQC spectral databases will experience an accelerating expansion as a result of SMART’s application.

The workflow (Fig. 1) of SMART begins with recording the NUS HSQC spectrum for a pure small organic molecule; in the case of NPR, this is a substance extracted and purified from an organism of interest, but just as easily could be a small molecule produced from organic synthesis, biosynthesis or from a metabolomic study. A small molecule is defined here as one whose transverse relaxation time constant (${T}_{2}$) is on the same order of magnitude as its longitudinal relaxation time constant (${T}_{1}$) when dissolved in liquid solution. In other words, the nuclear spins of a small molecule should be synchronized between 10^7^ to 10^8^ Larmor precession cycles during a liquid state 2D HSQC experiment^33^. Nevertheless, the SMART concept is not inherently confined to small molecule NUS NMR spectra, considering the ability of NMR to structurally characterize molecules of many sizes and types. NUS HSQC experiments are highly advantageous for small molecule structure elucidation compared with conventional pulse sequences due to their rapid acquisition, few spectral artefacts, and intrinsic high resolution. Nevertheless, as discussed below, conventional 2D HSQC spectra can be provided to the AI system and spectral recognition achieved. In fact, the initial database of HSQC spectra that were compiled to train the SMART system was acquired in this manner.

**Figure 2:**
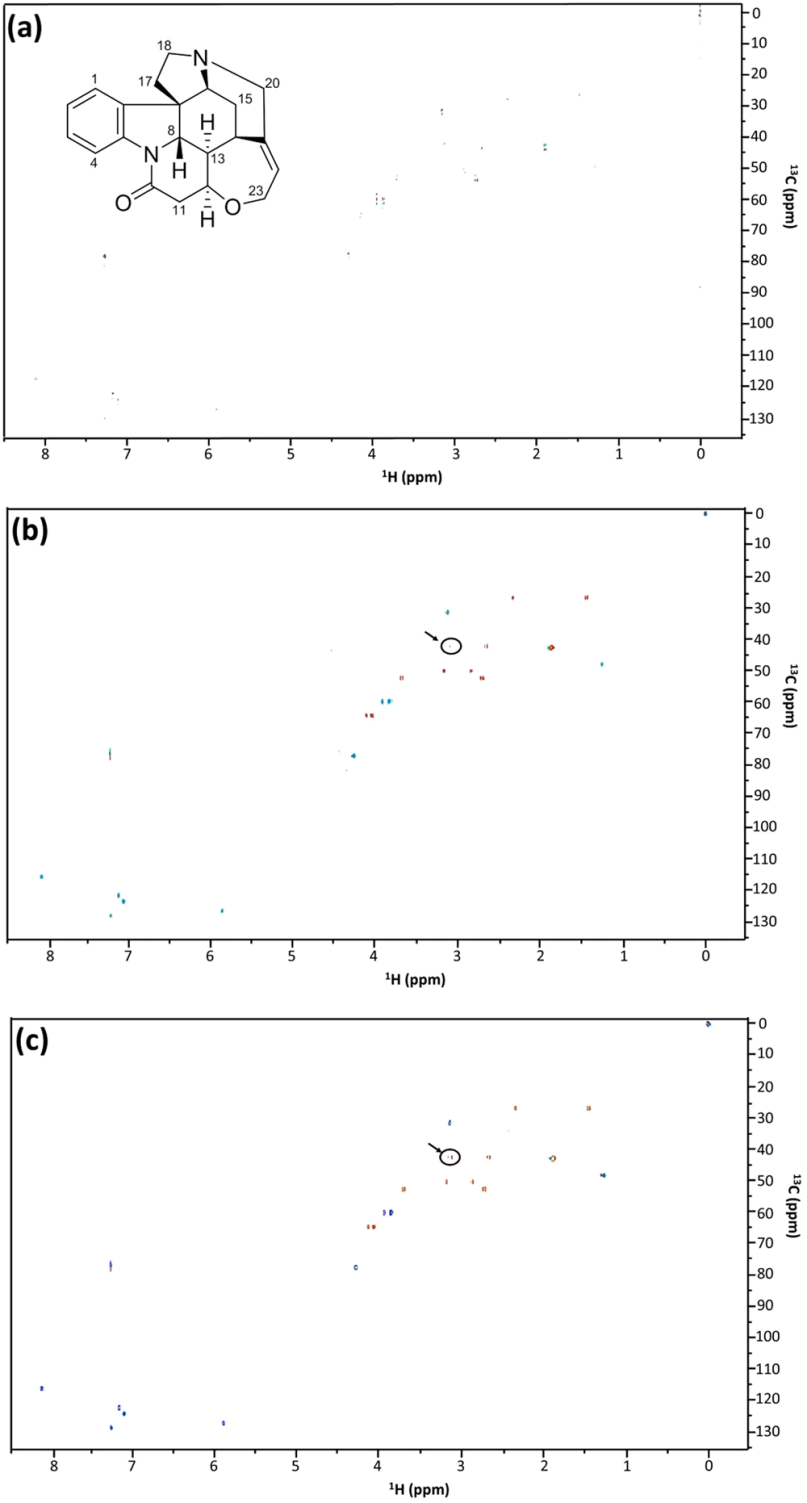
Data reconstruction results of a non-uniformly sampled (NUS) HSQC experiment. All of the three full HSQC spectra were recorded with a 50 nmole strychnine sample in CDCl_3_ on a 600 MHz Bruker 1.7 mm cryoprobe instrument, using 32 out of a total 128 increments (25% sampling density) in the indirect dimension and 8 scans. The differences between the three spectra were that (**a**) was transformed with the maximum entropy method (MEM) alone, (**b**) was transformed with the iterative soft thresholding (IST) alone, and (**c**) was transformed with IST followed by MEM. The doublet (see black arrows and circles in (**b**) and (**c**)) associates with the protons on the methylene (C-11) adjacent to the ketone in strychnine (see text for discussion).

Due to lower sampling density, NUS HSQC requires alternative approaches to convert the indirectly sampled time domain into visual spectra of the frequency domain, and thus methods other than the Discrete Fourier Transform are required. To this end, Iterated Soft Thresholding (IST)^34,35^ followed by the Maximum Entropy Method (MEM)^36,37^ was applied to NUS data collected for the model compound strychnine. In order to achieve convergence to a local minimum, a Lagrange multiplier was applied to weight the regularization function, the *L*_1_ norm, in the IST routine. Previous studies^12^ have shown that IST is superior to Maximum Entropy Reconstruction (MaxEnt)^38^ (not to be confused with MEM) in NUS NMR data reconstruction, owing to the simplicity of IST with fewer adjustable parameters and the resultant ease of application. Nevertheless, IST suffers slower convergence compared to MaxEnt for spectra with a high dynamic range. However, it has been shown that changing the step sizes in IST can achieve visualization of the final spectra indistinguishable from those reconstructed by a well-tuned MaxEnt process^39^. The MEM can then be applied after Fourier Transformation of the IST reconstructed data in the direct dimension, resulting in an improvement that derives from the fact that MEM is biased towards the enhancement of smaller line widths^40^. For the model compound, the HSQC correlation signals of the C-11 methylene protons (3.11 ppm and 2.67 ppm) to their subtending carbon were visibly strengthened after sequentially applying IST (400 iterations) and MEM (3 iterations) compared with application of IST (400 iterations) with Linear Predictions (LP) during data reconstruction of the non-uniformly sampled 2D NMR spectra (Fig. 2).

Our deep learning method is based on a siamese neural network architecture^41^. A siamese network is comprised of a pair of identical networks that are trained with pairs of inputs. These are mapped to a representational space where similar items are near one another and different items are further apart. As a result, it produces a clustering of the input space based on a similarity signal. In our case, it first maps the input HSQC spectra into a ten dimensional space, which then can be mapped into a two dimensional space by Principal Components Analysis (PCA) for visualization purposes.

**Figure 3:**
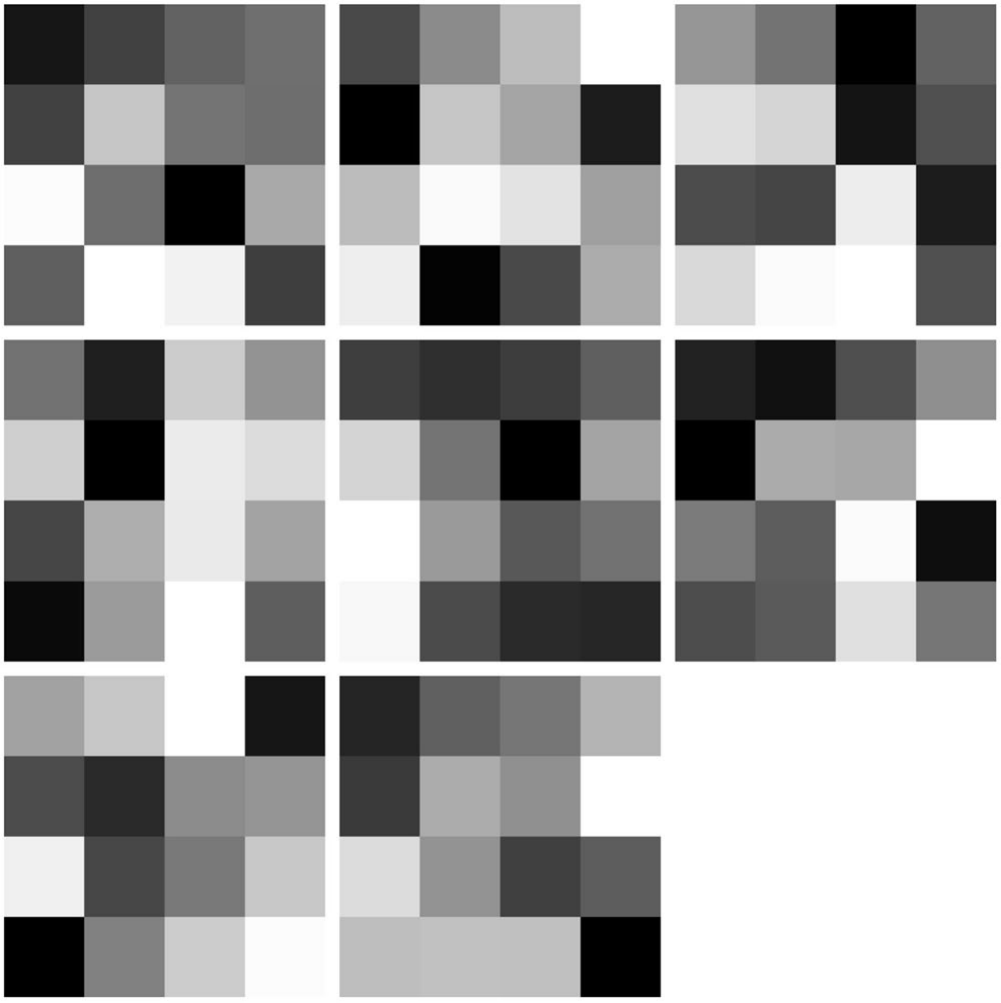
Features learnt by the first convolutional layer of the CNN. Feature maps were extracted from convolution layer 1 in Table 1, with the eight blocks of 4 × 4 pixels in this figure corresponding to the results of each of the eight filters applied to the HSQC dataset.

Because HSQC spectra are inherently a visual input, we used convolutional neural networks (CNNs)^42^ as the components of our siamese network. CNNs are currently the best method for image processing in the computer vision community, and have revolutionized the field of computer vision^42–44^. Like standard neural networks, they are trained by backpropagation of errors^45^. CNNs are structured to learn local visual features that are replicated across the input, hence the term “convolutional”. The local maximum of these features are then input to another layer that learns local features over the previous layer of features, and this process is repeated for several layers. In previous work, it has been shown that the feature maps resulting from each convolutional layer become more abstract as the layers of the network are traversed. We show the first layer features in Fig. 3. By using the local maxima of feature responses over nearby locations in the input, the network will generalize to patterns that are shifted in the $({f}_{1},{f}_{2})$ plane of the spectra, *i.e*., it achieves some translation invariance. Thus, the network is inherently hierarchical, like the mammalian visual system, and learns more and more abstract features in deeper layers of the network. In a siamese network, the final layer is not trained to classify the inputs; instead, a set of units are trained to give similar patterns of activation for similar inputs (as given in the teaching signal) and different patterns of activation for inputs that are labelled as different. Hence, they produce a clustering in the space of unit activations^46^.

As a result, molecules that are similar in HSQC spectra will be mapped to nearby locations in the output space. If the network generalizes well, it will place novel molecules near known ones that have similar NMR spectra. This allows the system to rapidly identify candidate known molecules that may have similar chemical features to the novel molecule, allowing the user to search through a small subset of known molecules for similar compounds. In our initial approach, we used ten output units (*i.e*., a 10 dimensional space), which can be visualized by applying Principal Components Analysis (PCA) to reduce the 10 dimensions to two.

### Network training and results

The neural network was trained using stochastic gradient descent^47^ with the Adagrad^48^ update rule. To speed the training, we employed batch normalization^49^, which reduces the internal covariance shift by standardizing the distribution of features on each layer. The network was found to train 7 times faster (wall clock time) using batch normalization.

When training the CNN, the datasets (see the Methods section for details) were divided into three subsets; the training set containing 80% of the spectra, used to adjust the parameters of the network, the validation set containing 10% of the data used for early stopping, and a test set containing the remaining 10% of the data (for details, see Methods). The test set consisted of HSQC spectra that were not used during the training process. The error from the validation set was monitored to prevent overfitting on unseen data. The test spectra were then embedded in the cluster map to locate their nearest neighbours. In this way, the test HSQC spectra were matched with other structurally similar compounds (*e.g*., from the same compound family or by possessing a high Tanimoto similarity score).

**Figure 4:**
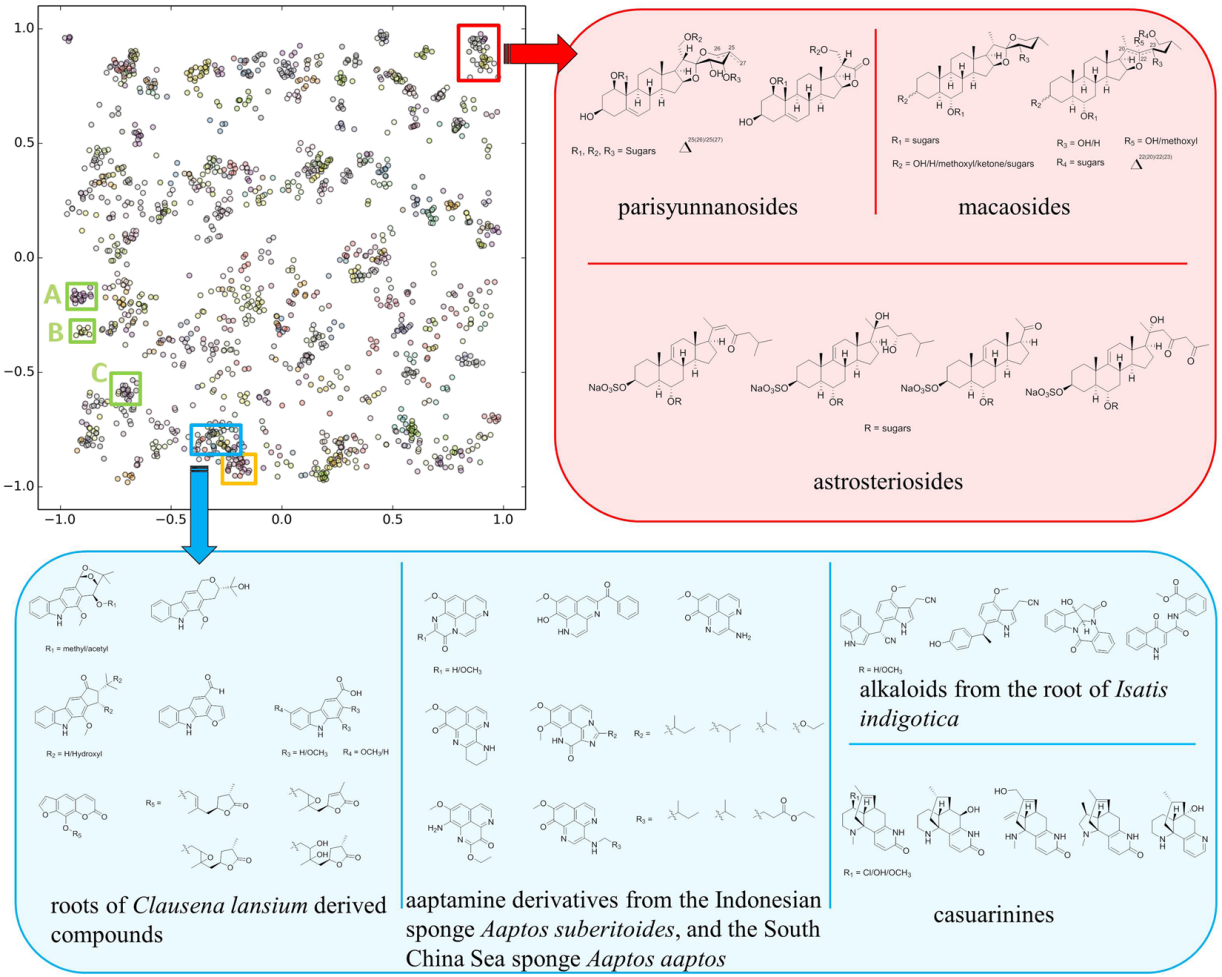
The SMART cluster map based on training result of 2,054 HSQC spectra over 83,000 iterations, with inset boxes representing different compound classes discussed in the text.

To produce visually comparable results, the outputs of both the training and the test sets in SMART were visualized in a two dimensional map (Fig. 4). Each node represents an HSQC spectrum processed by SMART. The node colours designate compounds originating from different research articles (e.g. usually different compound families). When available, the node labels are the compound names; otherwise, the labels are for the organism from which the compound derives. Here the dimension of embedding refers to the dimensions of the cluster space into which the siamese network maps the compounds. For example, if the siamese network had two outputs, we would be embedding the compounds into 2D. However, we have found that this is too restrictive, and does not perform well. Rather, in preliminary work we found that 10 dimensions provides optimal accuracy and precision-recall performance. Our illustrations in Fig. 4 are constructed by taking the 10D output of the network and applying PCA to map the 10D cluster space into 2D for illustration purposes. To evaluate the training algorithm, a smaller dataset containing 400 HSQC spectra was first mapped into node clusters with 4,800 training iterations (Figures S1 and S2 in the Supplementary Information for the cluster map with analysis), and subsequently, we trained on a larger dataset of 2,054 for a total of 83,000 iterations. The tight structural similarity between the compounds and their locations in the cluster map is evident (Fig. 4).

Structurally similar NPs were found to form distinct clusters in the map. Three clusters are discussed here to highlight this distinct clustering of different molecular entities, one for a terpenoid family, and two for aromatic alkaloid groups (Fig. 4). A cluster comprised of 40 nodes (red box, Fig. 4) was found to contain three saponin variants together with other corresponding triterpenoids. The three saponin variants, parisyunnanosides^50^, macaosides^51^, and astrosteriosides^52^, are of different geographic origins and are produced by organisms from different biological orders. The parisyunnanosides were isolated from the rhizomes of the terrestrial plant *Paris polyphylla Smith var. yunnanensis* originating in Lijiang, Yunnan Province, mainland China. The macaosides were obtained from the aerial parts of the terrestrial plant *Solanum macaonense* collected in Kaohsiung, Taiwan. Finally, the astrosteriosides were isolated from the marine starfish, *Astropecten monacanthus* found around Cát Bà island, Haiphong, Vietnam. The parisyunnanosides have been reported to be toxic to leukaemia cells^50^ whereas the macaosides and astrosteriosides have been found to be anti-inflammatory^51,52^. A second cluster consisting of 42 nodes (blue box, Fig. 4) was comprised of poly-heterocyclic aromatic alkaloids. Within this cluster there are four major molecular families (Table S1 in Supplementary Information) with the heterocyclic components being a pyrrole, imidazole, pyridine, or pyrazine, or a combination of these. Notably, several congeners of aaptamine, isolated from two varieties of *Aaptos* species collected in different geographic locations, are found in this cluster. A third cluster was composed of phenolic amides known as the teuvissides^53^ (orange box, Fig. 4); these latter compounds are reported to possess anti-hyperglycaemic properties and were isolated from *Teucrium viscidum* collected in Fujian Province, mainland China. The above discussion highlights the alternate basis for compound clustering by SMART as compared with geographical, pharmacological or source organism methods.

To explore the significance of cluster-to-cluster distance in the clustering map, we evaluated the types of structures present in three clusters that were well defined and in varying proximity to one another (green boxes A, B and C of Fig. 4). Cluster A was composed of oxidized steroids of highly similar structure to one another from the plants *Aphanamixis polystachya* ^54^ and *A. grandifolia* ^55^, whereas nearby cluster B was formed from a series of triterpene glycosides^56^. The more distant cluster C contained several diterpenoids^57^. Visually, it seems generally correct that oxidized steroids are more similar to triterpenes than they are to diterpenoids. In comparison, the averaged 2D Tanimoto score^58^ (a distance measure based on planar structures of compounds) between compounds in the cluster A and B, ${T}_{AB}=44$, slightly exceeded the value ${T}_{AC}=43$ between compounds in the cluster A and C (Figure S3 in the Supplementary Information for molecular structures), which indicates that the deep CNN method is better at quantifying and representing structural differences among compound subfamilies than the algorithm used to generate 2D Tanimoto scores. The average intra-cluster Tanimoto score of the cluster containing uralsaponins A, B, C, F, M, T, V, W, X and Y is 96.3 whereas the cluster containing aphanamixoids C, D, E, F and G is 95.7. The average intra-cluster Tanimoto score of the cluster containing ebractenoids A, B, C, D, E, F, G, H, I and J is 69.4. All of these intra-cluster Tanimoto scores are higher than the inter-cluster Tanimoto score ${T}_{AB}=44$ or ${T}_{AC}=43$. Therefore, it is apparent that the SMART clustering map not only recognizes closely similar compounds, but also appropriately places clusters of different compounds in their proper context relative to one another.

### Related work

Again, the aforementioned grid-cell approaches^28^ are similar to ours in that the shifted grid positions can be thought of as corresponding to the first layer of convolutions, which have small receptive fields (like grid cells), and they are shifted across the input space like shifted grids. Also, our approach uses layers of convolutions that can capture multi-scale similarities. The grid-cell approaches, however, use hand-designed features (i.e. counts of peaks within each grid cell), and the similarities are computed by simple distance measures. In particular, PLSI and LSR are linear techniques applied to hand-designed features. Furthermore, other representations, for example the ‘tree-based’ method^59^, also rely on data structures designed by the researcher. Our approach, using deep networks and gradient descent, allows higher-level and nonlinear features to be learned in the service of the task. This approach is similar to modern approaches for computer vision, which since 2012 has shifted away from hand-designed features to deep networks and learned features, and has led to orders of magnitude better performance. Similarly to how deep networks applied to computer vision tasks have learned to deal with common problems, such as recognizing objects and faces in different lighting conditions and poses, our CNN pattern recognition-based method can overcome solvent effects, instrumental artefacts, and weak signal issues.

**Figure 5:**
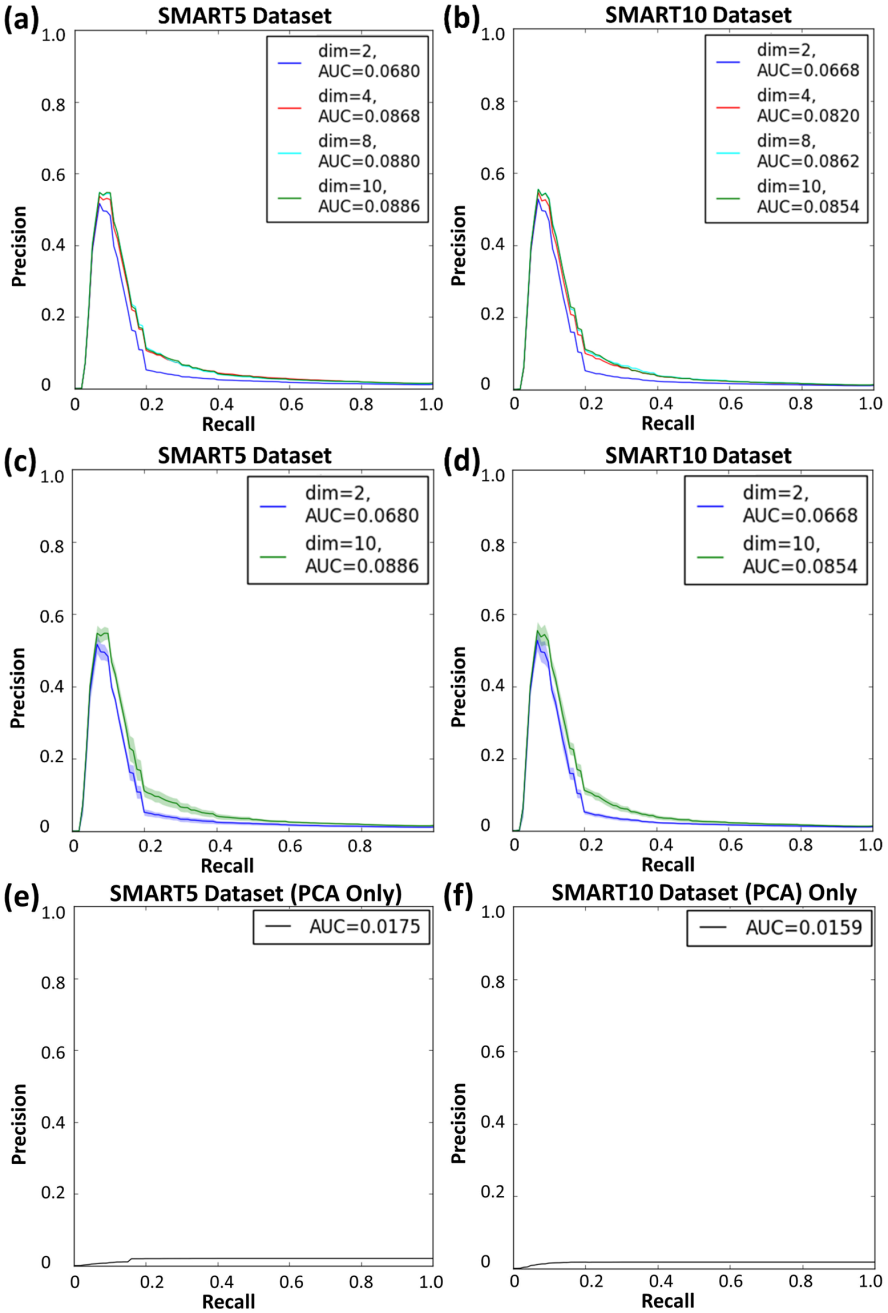
Precision-recall curves measured across 10-fold validation for different dimensions (dim) of embeddings. (**a**) and (**b**) Mean precision-recall curves on test HSQC spectra for SMART5 and SMART10 datasets, respectively. (**c**) and (**d**) Mean precision-recall with error curves (grey) for SMART5 and SMART10, respectively. (**e**) and (**f**) Mean precision-recall curves for SMART5 and SMART10 clustered by Principal Component Analysis (PCA) without use of the CNN. AUC: area under the curve.

**Figure 6:**
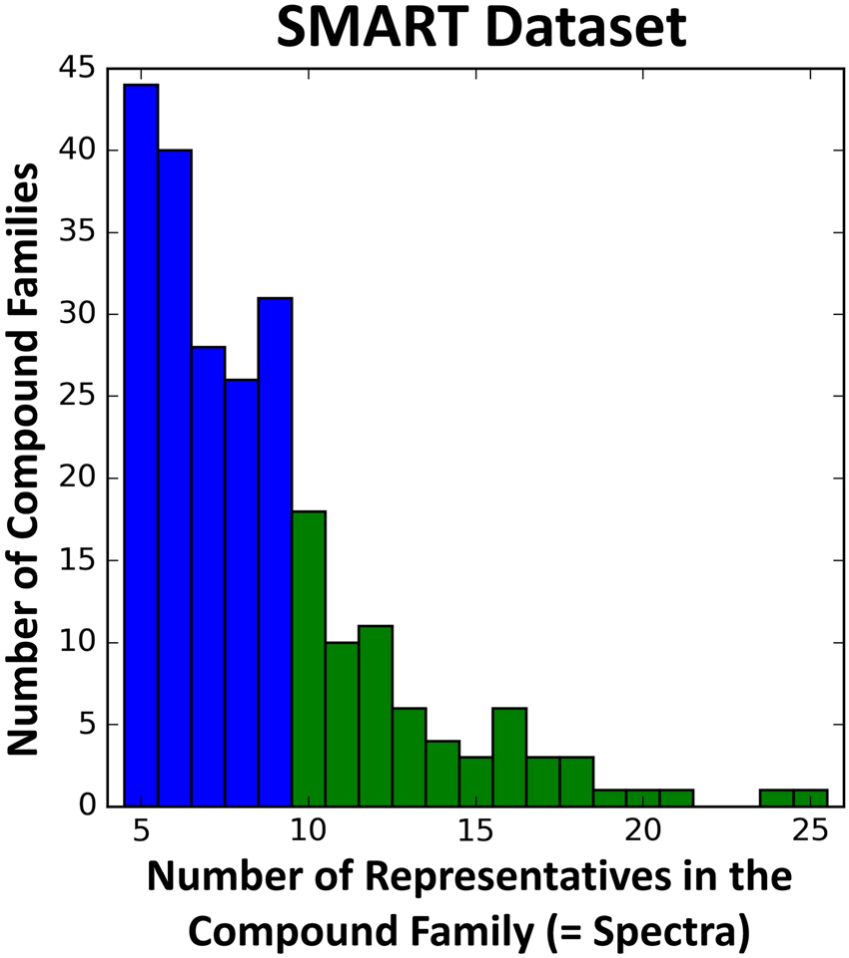
Distribution in the Training Dataset of Numbers of Families Containing Different Numbers of Individual Compounds. The SMART5 training set contains 238 compound subfamilies, giving rise to 2,054 HSQC spectra in total. (Blue and Green) The SMART10 training set contains 69 compound subfamilies and is composed of 911 HSQC spectra in total. (Green only).

It is difficult to directly compare Wolfram *et al*.’s results to ours because they used a much smaller dataset (132 compounds) from 10 well-separated families. This is not enough data to train the deep network. To further compare our approach with other NMR pattern recognition approaches, we generated precision-recall curves (Fig. 5) using SMART trained with the SMART5 and SMART10 databases (Fig. 6). Considering SMART as a search engine, precision recall curves help evaluate the SMART’s performance to find the most relevant chemical structures, while taking into account the non-relevant compounds that are retrieved. In our approach to HSQC spectra recognition/retrieval, precision is a measure of the percentage of correct compounds over the total number retrieved, while recall is the percentage of the total number of relevant compounds. Therefore, higher precision indicates a lower false positive rate, and higher recall indicates a lower false negative rate. The precision-recall curves of our approach show high precision peaks at low recall rates, suggesting that SMART retrieves at least some relevant structures in the first 10–20% of compounds retrieved, and thus indicates that SMART returns accurate chemical structures. To compare this to a linear embedding, we performed PCA on the SMART5 and SMART10 databases separately. The precision recall curves of those PCA results are much worse than those processed by the CNN (Fig. 5).

### SMART recognition of noisy HSQC spectra

**Figure 7:**
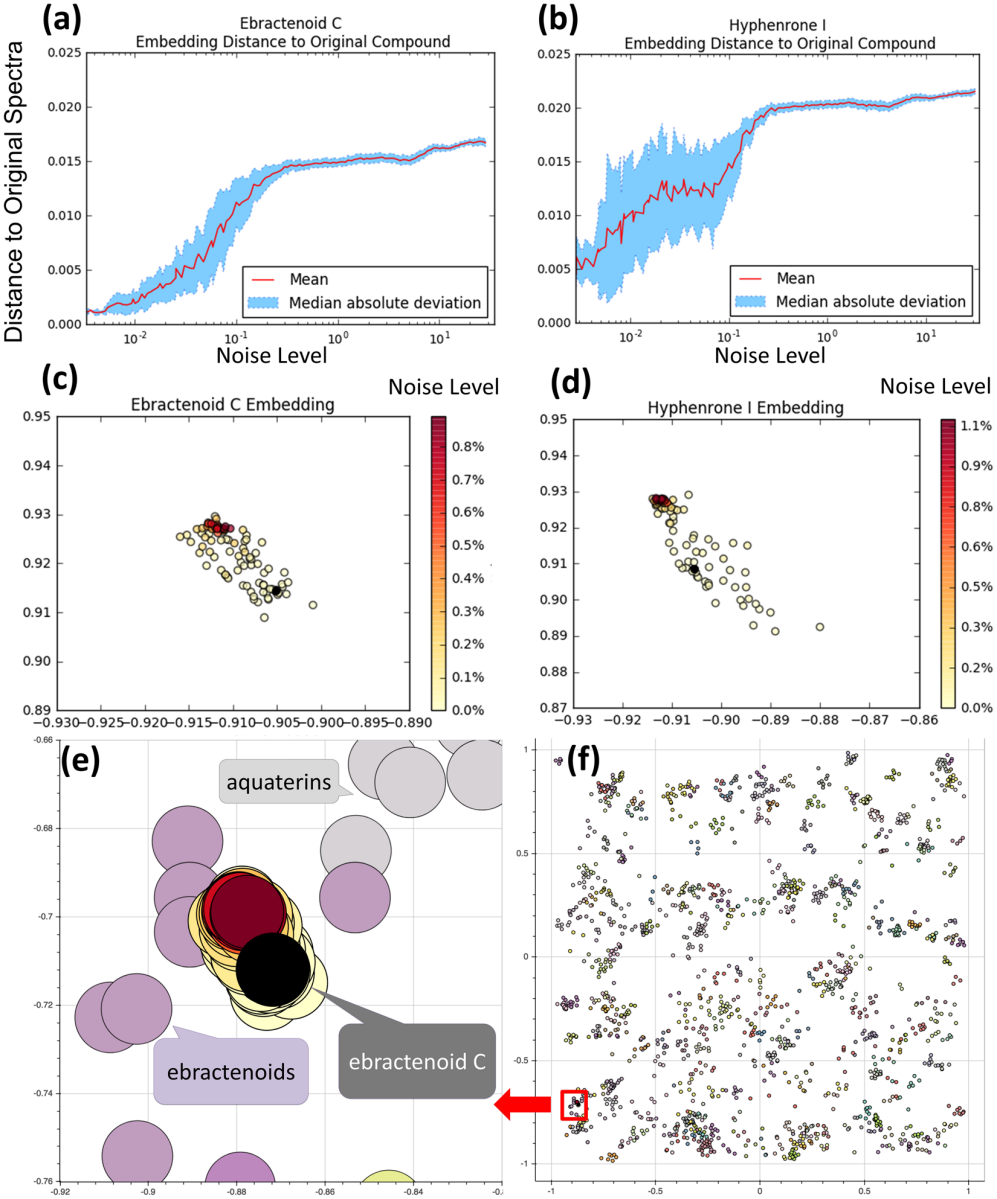
Distance of the noisy spectra measured against the original spectra of ebractenoid C and hyphenrone I. The distance measure in the y axis of the ebractenoid plot (**a**) and hyphenrone plot (**b**) is the same as the cluster map in Figs 4 and 7(f). The noise level is defined by dividing pixels altered over the total number of pixels of the HSQC spectra. The results visualized in the 2D cluster maps with each node representing one noisy spectra, and with node color intensity as a function of the noise level, for the ebractenoids (**c**) and hyphenrones (**d**). The original image without added noise is shown as the black node in these 2D cluster maps. We then embedded the nodes for the ebractenoids in (**c**) to a global view of the 2D cluster map in (**f**), and zoom in on the red box in (**f**) as shown in (**e**). Note, larger node sizes are used to depict compounds in (**e**) versus (**c**).

Because white Gaussian noise is often seen in experimental HSQC spectra, we investigated the robustness of the SMART to recognize HSQC spectra in the presence of significant noise. By adding noise to HSQC spectra in the SMART10 database and measuring the Euclidean distance of those noisy spectra to their original ones, we were able to observe that as noise intensity increases so does the distance increase from the original location in the 2D cluster map. However, the noisy spectra were still effectively recognized as being closely related to their original compounds (Fig. 7 and Supplementary Information).

### SMART characterization of Viequeamides of NRPS origin

As a practical example of the functional use of the SMART workflow to discover new NPs, we used it to rapidly characterize a group of unknown marine cyclic depsipeptides from two marine cyanobacteria: 1) *Rivularia sp*. collected in Vieques, Puerto Rico and 2) *Moorea sp*. collected in American Samoa. These compounds were isolated in the course of our ongoing efforts to discover marine cyanobacterial NPs with anti-cancer properties^60^. Metabolites from these two collections were purified by high performance liquid chromatography (HPLC), and then ^1^H-^13^C HSQC data were collected with 100% sampling density, but using the NUS pulse sequence in the indirect dimension for all six purified compounds. Data reconstruction as described above for the six samples yielded HSQC spectra, and these were subjected to the SMART workflow to embed them in the cluster map. We found that the six nodes clustered with nodes for the previously characterized viequeamides A (**1**) and viequeamides B (**4**). After an analysis of various 2D NMR spectra, and MS, IR and UV data, the planar structures of the four new compounds were determined (Fig. 1, compounds **2**, **3**, **5**, **6**). The absolute configurations of these compounds were then elucidated by Marfey’s analysis and/or X-ray crystallography, completing their structure determination. Evaluation of the toxicity of the pure compounds to H-460 human lung cancer cells revealed that two possessed relatively potent cancer cell toxicity properties; viequeamide A2 (**2**) had an IC_50_ = 0.62 μM and viequeamide A3 (**3**) had an IC_50_ = 1.98 μM. Viequeamides B (**4**), C (**5**) and D (**6**) showed no appreciable H-460 human lung cancer cytotoxicity.

## Methods

### Training set collection and processing

The dataset of HSQC spectra was compiled from available online sources. We removed spectra that showed no peaks (*i.e*., the spectra in the publication appeared blank). We collected all usable^1^H-^13^C HSQC spectra (4,105 in total), including a few cases of the same compound in different deuterated solvents, from the supporting information of the *Journal of Natural Products*, years 2011, 2012, 2013, 2014 and 2015. In addition, the HSQC spectra of somocystinamide A^61^ and swinholide A^62^ in the supporting information of *Organic Letters* were also included in the dataset. Around 2,056 spectra were removed from this series, because their molecular class had less than 5 HSQC spectra. All spectra were collected and initially processed by the following steps: (1) The HSQC spectra were saved as png format grayscale images at a resolution of 512 × 512 pixels (the minimum resolution in the proton dimension is 51.2 pixels per ppm and in the ^13^C dimension it is 2.8 pixels per ppm.); (2) lines surrounding spectral edges, annotations, chemical structures, and other annotations were deleted using Photoshop such that only the HSQC signals and noise were present in the images; (3) images were rotated and/or flipped when necessary to ensure that the horizontal dimension was the direct^1^H dimension with chemical shifts increasing from right to left, and the vertical dimension was the indirect^13^C dimension with chemical shifts increasing from top to bottom; (4) images were uniformly converted into black (signal and noise) and white (spectral background); (5) images from the same publication were pooled and labelled as the same training class, as all of the publications we used reported compounds from a single family; (6) a cross shaped 3 × 3 median filter^63^ was applied to remove unwanted salt-and-pepper noise; however, no other enhancements were applied (Figure S4 in the Supplementary Information for an example of spectra input preparation). Essentially, in this study, the relevant quantity for measuring similarity was the positions and shapes of the various peaks relative to one another, rather than their absolute positions.

Figure 6 shows the distribution of spectra number within each compound family in the complete dataset. From Fig. 6, we observe that the dataset has a skewed distribution of images per class. Hence, in order to make the training stable and comparison fair, we created two different datasets: SMART5 and SMART10, containing all spectra of compound families (*e.g*. veraguamides^64^, ebractenoids^57^, naphthomycins^65^, viequeamides, etc.) with at least 5 and 10 HSQC spectra, respectively, per family. In total we have 238 categories (2,054 spectra) for SMART5, the largest having 25 and the lowest having 5 spectra per compound family. Further restricting the data to contain at least 10 spectra per molecular class results in only 69 categories (911 spectra) in SMART10, which we found to be too few for effective training. Hence, all of our experiments used SMART5.

When training the neural network (see below for description), we used a 10-fold cross-validation scheme, randomly shuffling the dataset and then splitting it into training, validation, and test sets in proportions 8:1:1. We repeated the procedure 10 times such that all images became part of a test set one time. The results we report here were averaged across these ten networks

### NUS 2D NMR data generation

In order to generate an independent test set, we developed an optimized NUS pulse sequence using an NMR standard (strychnine, 50 nmole TCI America, Catalog No. S0249). This optimized method was then applied to several newly isolated NPs (e.g., the viequeamides). The viequeamides were isolated from two different marine cyanobacteria; *Rivularia sp*. collected in Vieques, Puerto Rico^60^ and *Moorea sp*. collected in American Samoa. Detailed isolation and structural elucidation of these compounds will be published separately. The 2D NMR spectra were recorded on a 600 MHz Bruker Avance III spectrometer with a 1.7 mm Bruker TXI MicroCryoProbe^TM^ using TopSpin 2.1. The solvent CDCl_3_ contained 0.03% v/v trimethylsilane (δ_H_ 0.0 and δ_C_ 77.16 as internal standards using trimethylsilane and CDCl_3_, respectively). All spectra were recorded with the sample temperature at 298 °K.

The data shown in Fig. 2 were acquired using the NUS edited hsqcedetgpsisp2.3 HSQC pulse sequence. Data were acquired as 4096 × 32 points (32 out of 128 t_1_ increments, 25% NUS) in direct and indirect dimensions, respectively, giving a total acquisition time of a quarter of its conventional counterpart. Spectral windows in direct and indirect dimensions were 7183.9 and 24118.9 Hz respectively. Data in both Fig. 2(b) and (c) were processed using NMRPipe^66^ by applying zero filling (round final size to power of 2) in both dimensions. Spectra in Fig. 2(b) were processed by applying IST as implemented in hmsIST^12^ with 400 iterations followed by forward-backward LP sequentially, while spectra in Fig. 2(c) were processed by applying IST with 400 iterations followed by MEM with the standard deviation of time-domain noise set to 200. The viequeamides spectra were acquired and processed the same way as Fig. 2(c), except that the indirect dimension was sampled with 100% NUS (256 out of 256 t_1_ increments).

### The deep siamese network

As depicted in Table 1, the overall deep CNN siamese architecture used in this study is similar to AlexNet^42^, and consists of 8 layers comprised of 4 convolutional layers followed by 4 fully connected layers. This network is used as the two “twins” in the siamese network. The output layer contains vectors in ${{\mathbb{R}}}^{K}$. Here, *K* is the embedding dimension. The energy loss function defined in equation 2 (below) is applied to the outputs of the embedding layer (layer 8). We ran several experiments to find the best *K* and measured the accuracy on the validation set. Empirically, for the given dataset, $K=10$ gave us the best results.

**Table 1:** The Architecture of the Deep CNN Used in This Study^a^.

Layer Number	Layer Type	Number of Filters (Stride 1)	Size	Additional Information
1.	convolutional	8	4 × 4	maxpool 4 × 4 stride 2
2.	convolutional	16	4 × 4	maxpool 4 × 4 stride 2
3.	convolutional	16	4 × 4	maxpool 4 × 4 stride 2
4.	convolutional	16	4 × 4	maxpool 4 × 4 stride 2
5.	fully connected	—	128	dropout 0.5
6.	fully connected	—	128	dropout 0.5
7.	fully connected	—	128	dropout 0.5
8.	fully connected	—	*K*	*K*-dimensional embedding layer

^a^The dimensionality of the input data is 512 × 512.

### Loss function

Siamese networks are trained with an energy function that is minimized by gradient descent. The design of the energy function determines the way in which pairs of items are pushed together or pulled apart. There are at least two such functions that have been used^30^ in the literature; here, we used a modified version of the spring model developed by Hadsell *et al*.^41^. The energy function is described with the following notation; for example *i*, the input vector is represented as *x*_*i*_, and the output label as *y*_*i*_. The output label is defined as a “one hot” vector, where if there are *k* categories, *y*_*i*_ is a *k*-dimensional binary vector, and if the category is *c, y*_*i*_ is 1 at the *c* ^*th*^ position and 0 everywhere else. Meanwhile, *x*_*i*_, the input HSQC spectra, is treated as a vector.

We treat our neural network as a function *G*_*W*_, where *W* is the weights of the network. Then the output of the neural network is ${G}_{W}(x)$. ${G}_{W}(x)$ is a vector of dimension *K*, a hyperparameter of the system. We then define the distance function *d* between images ${x}_{i}$ and ${x}_{j}$:

1 $${\boldsymbol{d}}({{\boldsymbol{x}}}_{{\boldsymbol{i}}},\,{{\boldsymbol{x}}}_{{\boldsymbol{j}}})=\Vert {{\boldsymbol{G}}}_{{\boldsymbol{W}}}({{\boldsymbol{x}}}_{{\boldsymbol{i}}})-{{\boldsymbol{G}}}_{{\boldsymbol{W}}}({{\boldsymbol{x}}}_{{\boldsymbol{j}}})\Vert $$

where $\Vert \cdot \Vert$ is the Euclidean distance function.

Now we can define the energy function *L* to be minimized as^41^:

2 $${\boldsymbol{L}}({{\boldsymbol{x}}}_{{\boldsymbol{i}}},\,{{\boldsymbol{x}}}_{{\boldsymbol{j}}})=\{\begin{array}{c}\frac{1}{2}\,{\bf{\max }}\,{(0,{\boldsymbol{d}}({{\boldsymbol{x}}}_{{\boldsymbol{i}}},{{\boldsymbol{x}}}_{{\boldsymbol{j}}})-{\boldsymbol{m}})}^{2},{\boldsymbol{if}}\,{{\boldsymbol{y}}}_{{\boldsymbol{i}}}={{\boldsymbol{y}}}_{{\boldsymbol{j}}}\\ \,\frac{1}{2}\,{\bf{\max }}\,{(0,{\boldsymbol{m}}-{\boldsymbol{d}}({{\boldsymbol{x}}}_{{\boldsymbol{i}}},{{\boldsymbol{x}}}_{{\boldsymbol{j}}}))}^{2},{\boldsymbol{otherwise}}\end{array}\,$$

where $m$ is a hyperparameter that defines a margin. In this case, if ${y}_{i}$ and ${y}_{j}$ are the same category and the squared distance between the output representations of *x*_*i*_ and ${x}_{j}$ is more than a margin, then this distance is minimized, otherwise it is unchanged. If they are different, then we should increase the distance between them up to the margin *m*. Once they are pushed at least m distance apart, the loss becomes 0. This loss function penalizes large distances between pairs of outputs for images in the same category (first line), but for outputs from different categories, a penalty is assigned only if they are within $m$ units. This loss function ensures that the output space forms well-behaved clusters during training. The difference between this objective function and the one used in Hadsell *et al*.^41^. is that no margin was used within the same category. Empirically, we find this objective function gives superior results.

### Training details of the siamese network

We implemented our system using the Theano^67^ and Lasagne (http://tinyurl.com/hl9dy9y) deep network packages. The siamese network was trained using mini-batch stochastic gradient descent with the Adagrad^45^ update rule, following the protocol introduced by Hadsell *et al*.^41^. Specifically, 50% of each mini-batch has negative samples $(({x}_{i},\,{x}_{j},{y}_{i},{y}_{j})$ s.t. $({y}_{i}\ne {y}_{j}))$, and 50% has positive samples $(({x}_{i},\,{x}_{j},{y}_{i},{y}_{j})\,{\rm{s}}{\rm{.t}}{\rm{.}}({y}_{i}={y}_{j}))$. The Adagrad update rule tunes the step size automatically in real time, making learning stable in later iterations. We used hyperbolic tangent as the activation function for all layers including the output layer. The weights were initialized using Xavier initialization^68^. The initial learning rate was $\alpha =0.001$, and the mini-batch size was 256. We applied dropout regularization^69^ on layers 5, 6, and 7 of the network, and batch normalization^49^. We found that applying batch normalization speeds convergence by a factor of 7. The total number of parameters in the network is 399,102, considering that the number of parameters triples when batch normalization is applied. We used Amazon EC2 instances to run our experiments.

We recorded precision-recall curves (Fig. 5) of SMART’s performance by randomly selecting HSQC spectra from the test dataset and retrieving known compounds according to their distance to the test compound in the cluster map. In this regard, precision was calculated by dividing the number of true positives over the combination of the number of true positives and the number of false positives. Recall was calculated as the number of true positives over the combination of the number of true positives plus the number of false negatives. At each level of recall, there is a different level of precision. The area under the precision recall curve (AUC) is then a standard measure of performance (larger is better). In our case, for each compound in the test dataset, we measured a precision recall curve by calculating precision (the number of retrieved compounds that are relevant) and recall (the number of relevant compounds that are retrieved) of the retrieved HSQC spectra from the training dataset within an expanding hypersphere centred at the compound in the test dataset. These final precision recall curves were averaged over the test dataset. The CNNs that we used in this regard were trained for 10,000 iterations on the SMART5 and SMART10 datasets with 10-fold cross validation for embedding dimensions k = 2, 4, 8, and 10 (Fig. 5). To compare our results to a linear embedding, we separately performed PCA on the SMART5 and SMART10 databases. Specifically, we embedded the PCA results in high dimensional Euclidean space (k = 10, chosen to match the number of dimensions used in the CNN training). The precision recall curves of the randomized results are also shown in Fig. 5.

**Figure 8:**
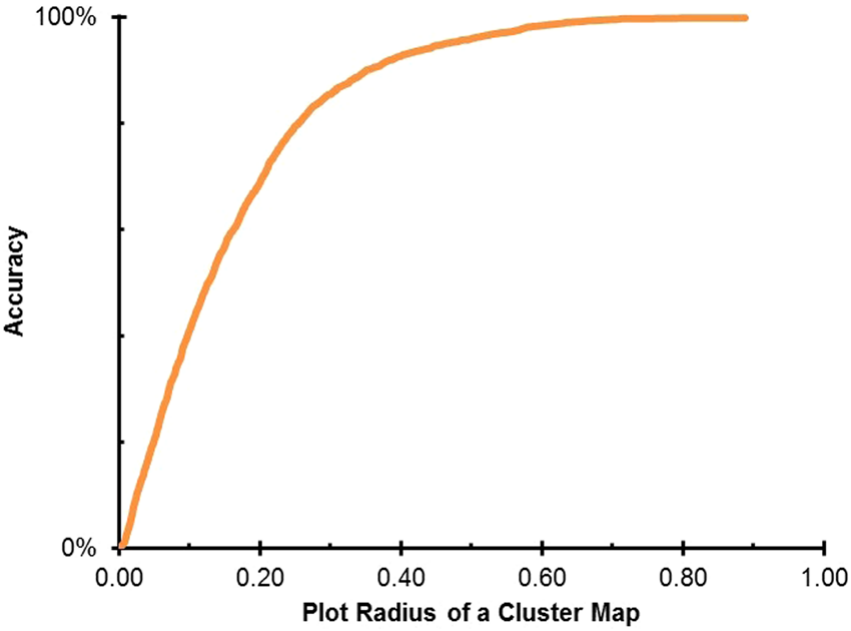
Plot of the Accuracy of SMART as the radius around a project point increases. This figure shows the fraction of correct families captured by a hypersphere of the given radius around each node in the cluster map. The distances between nodes in the cluster map has no physical meaning, but is a quantification of HSQC spectral similarity. SMART can achieve good accuracy (proper placement in the map of a new compound to its correct compound family) within 0.5 radius of a 2-dimensional cluster map, and even better for a 10-dimensional map.

**Figure 9:**
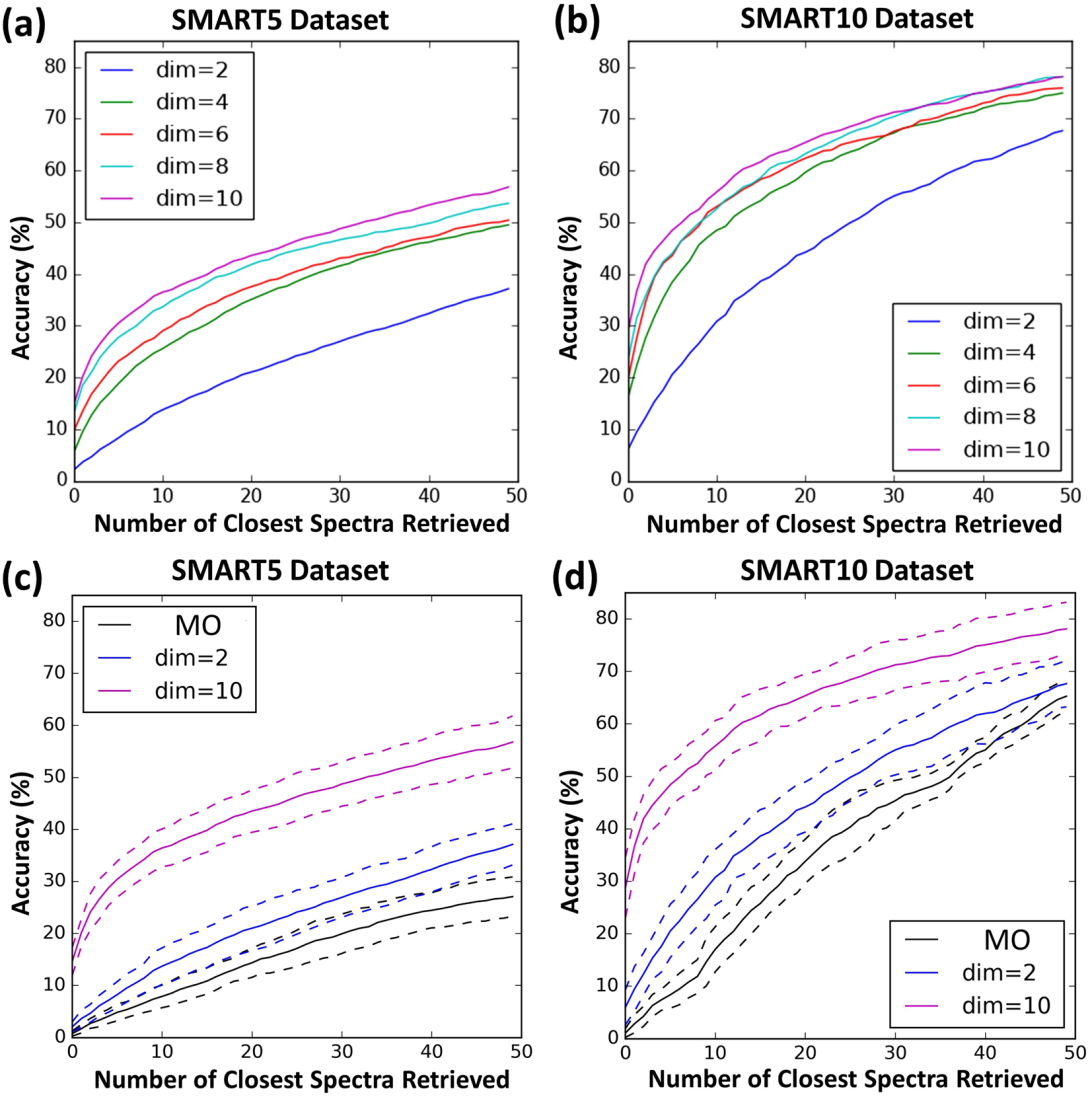
Closest retrieval curves measured across 10-fold validation for different dimensions (dim) of embeddings. For (**a**) and (**b**), mean closest retrieval curves on test sets for SMART5 and SMART10 datasets, respectively. For (**c**) and (**d**), mean closest retrieval curves with error curves (${\boldsymbol{\mu }}\pm {\boldsymbol{\sigma }}$, dashed lines) for SMART5 and SMART10, respectively. In (**c**) and (**d**), the black plot (MO, most frequently occurring) is a baseline prediction of random compound associations on the basis of the number of members in a compound family. Specifically, the category with the most members is picked as the first compound association, the second most members as the second one, etc. This order is the same irrespective of the compound being considered.

We also used 10-fold cross validation to estimate performance (Figs 8 and 9). Specifically, a different 10% of the training set was held out as a test set 10 times, and the results were averaged to report performance. For each fold of the cross validation, we held out 10% of the data for early stopping. In this way, all of our HSQC spectra were used for testing. Here, the complete split was 8:1:1, training:validation:test. The iterations stop at the point in training where the error on the hold-out set is minimized. Here, the error was a measure of average precision on the hold-out set.

We employed the Tensorflow package (https://tinyurl.com/y9lz45sa) to visualize the features that were learnt by the first layers of the CNN. The results of the first convolutional layer are shown in Fig. 3.

### Validation of the model on “novel” categories

To evaluate whether the system performs properly with new categories of molecules, we performed the following three experiments. In SMART5, we removed the HSQC spectra of three categories of compounds (ebractenoids, naphthomycins, and veraguamides) from each of three common NP families (terpenoids, polyketides, and peptides, respectively), for each experiment, and used those removed spectra as a test set. During training, each subfamily was given a different label, however, this information was only provided to the training algorithm in the sense of “same/different category” as in Equation 2. This experiment thus tested whether a subfamily of terpenoids that was unfamiliar to the network would be mapped close to the other terpenoids. For example, there are 10 compounds in the terpenoid subfamily of ebractenoids that were not used during training. During testing, they were presented to the network, and their distance to the other terpenoids measured. This experiment was repeated for the naphthomycins, and veraguamides, and their location in the embedding space was evaluated for whether they were properly mapped to their respective families (*e.g*. polyketides and peptides, respectively). This experiment revealed that the ebractenoids clustered with the terpenes and terpenoids in the 10-dimensional space (Table S2). Similarly, the naphthomycins and veraguamides were subjected to a similar experiment (Table S3,S4) and confirmed that SMART was able to properly place compounds to which it was naive.

Finally, we trained the siamese network using all of SMART5, supplemented with HSQC spectra for viequeamide A (**1**) and B (**4**) (2 spectra), parguerene, precarriebowmide, palmyrolide and three isomers (4 spectra), somocystinamide and a derivative (2 spectra), and columbamides A, B and C (3 spectra). This was exposed to the six newly collected HSQC spectra [subsequently identified as the viequeamides, e.g., the two known viequeamides A (**1**) and B (**4**) and four new viequeamides A2 (**2**), A3 (**3**), C (**5**) and D (**6**)] using the 100% NUS sampling method. Training was stopped after a fixed number of iterations. The 10-dimensional output of this test is presented in the Supplementary Information (Table S5).

### Tanimoto score calculation

Averaged Tanimoto Score for compounds between the three clusters in Fig. 1 was calculated using the PubChem Score Matrix Service^70^.

### Recognition of noisy HSQC spectra

Using Matlab 2013, we created a 2D matrix of white Gaussian noise to simulate the noise in real-time measurements. Next, we applied 2D Fast Fourier Transform (FFT) to this 2D noise matrix. The transformed FFT results for these noisy spectra were sized to match those of two randomly selected compounds (hyphenrone I and ebractenoid C) from the SMART10 database^57,71^. We also calculated the noise intensity in the spectra by dividing the number of noisy pixels by the total number of pixels. The noise matrix was then added to the two HSQC spectra, and the intensity of the noise was then increased consecutively in a finite arithmetic progression of 140 steps, rendering 140 noisy spectra for each compound. In addition, at each noise level, the white noise was again randomized 100 times, rendering a total of 14,000 noisy spectra. These noisy HSQC data were then processed by the convolutional neural networks pre-trained with SMART10 for over 10,000 iterations. The results are shown as two distance vs. noise plots in Fig. 7(a) and (b). The distance measure displayed in the vertical axis of these two plots was in the same units as the cluster map in Fig. 4. The results were also visualized in 2D cluster maps with each node representing one noisy spectrum, with the intensity of the node color representing the noise level (Fig. 7(c) and (d)). The original image without added noise is shown as the black node in those 2D cluster maps. In order to further visualize the internode distance between nodes that represent noisy spectra and those that represent our training dataset, we embedded the nodes of the noisy spectra in Fig. 7(c) in a global view of the 2D cluster map shown in Fig. 7(f), and provided a zoomed-in view of the ebractenoids clusters in Fig. 7(e). Figure 7(e) shows that noisy HSQC spectra are clustered close to their original spectrum, and thus, noise to the levels we have evaluated, has little effect on the ability of SMART to accurately place compounds into their appropriate location (ebractenoids in this case). Selected noise maps are provided in the Supplementary Information.”

## Conclusions and Future Work

SMART is the first combination of NUS 2D NMR and deep CNNs. This tool streamlines dereplication and determination of natural product families from multiple organisms and facilitates their isolation and structural elucidation. While compound families represented the metadata associated with HSQC spectra in this study, it is very possible to associate and integrate biological, pharmacological and ecological data with SMART, and thereby create new tools for enhanced discovery and development of biological active NPs as well as other small molecules. Ultimately, this leads to an increased appreciation for the structural diversity and therapeutic potential of natural products.

By both quantitative and qualitative inspection of SMART's cluster space, the following properties were suggested by the results: 1) the distance between nearby nodes of a clustering map is a measure of the structural similarity between compounds that share molecular moieties (e.g., functional groups, carbon skeletons, etc.), 2) chimeric compounds with structural features comprised of two independent families of compounds reside near or in between the component clusters (for example, saponins are located near and between other glycosides and terpenoids, in Fig. S2), 3) this accuracy of placement of new compounds in SMART should be enhanced as the size of the training set grows, 4) as the size of the training set increases for a given compound class, the accuracy of placement of a new test compound in that family improves, 5) even in the presence of random spectral noise, spectra are strongly associated to their structural chemical analogues. Nevertheless, the accuracy of recognition correlating to the signal-to-noise ratio of HSQC spectra remains to be determined, as does the impact of solvent effects on chemical shifts or extraneous peaks appearing in the spectrum from electronic sources or impurities. As more compounds are added to the training set, the SMART system will naturally improve in accuracy and robustness, thereby accelerating natural product structural elucidation and thus drug discovery.

SMART has an immediate value in NP drug discovery efforts by providing rapid and automatic compound dereplication and assignment to molecular structure families. With further refinement of the SMART workflow, such as training for spectra of the same compound with different S/N ratios, deeper understanding of other parameters that impact spectral recognition, combining with other fast NMR techniques, SMART has the potential to enhance NPR and enable new directions of experimentation at the chemistry-biology interface.

## Electronic supplementary material

Supplementary Information
