# Author Correction: Small Molecule Accurate Recognition Technology (SMART) to Enhance Natural Products Research

**DOI:** 10.1038/s41598-020-60108-0

**Published:** 2020-03-10

**Authors:** Chen Zhang, Yerlan Idelbayev, Nicholas Roberts, Yiwen Tao, Yashwanth Nannapaneni, Brendan M. Duggan, Jie Min, Eugene C. Lin, Erik C. Gerwick, Garrison W. Cottrell, William H. Gerwick

**Affiliations:** 1 Department of Nanoengineering, University of California, San Diego, La Jolla, California 92093 United States of America; 2 Department of Computer Science and Engineering, University of California, San Diego, La Jolla, California 92093 United States of America; 3 Center for Marine Biotechnology and Biomedicine, Scripps Institution of Oceanography, La Jolla, California 92037 United States of America; 4 School of Pharmaceutical Sciences, Guangzhou Medical University, Guangzhou, Guangdong 511436 People’s Republic of China; 5 Skaggs School of Pharmacy and Pharmaceutical Sciences, University of California, San Diego, La Jolla, California 92093 United States of America; 6 Department of Electrical and Computer Engineering, University of California, San Diego, La Jolla, California 92093 United States of America; 7 Vanderbilt University Institute of Imaging Science, Vanderbilt University, Nashville, Tennessee 37235 United States of America; 8 Department of Radiology and Radiological Sciences, Vanderbilt University, Nashville, Tennessee 37235 United States of America; 9 Physikalisches Institut, Universität Göttingen, Friedrich-Hund-Platz 1, 37077 Göttingen, Germany

Correction to: *Scientific Reports* 10.1038/s41598-017-13923-x, published online 27 October 2017

In this Article, the precision-recall curves in Figure 5 were computed incorrectly due to an error in the code that used an incorrect relationship between the recall and precision points. The new Figure uses the scikit-learn^1^ routine for computing precision-recall curves.

The correct Figure 5 appears below as Figure 1.

**Figure 1:**
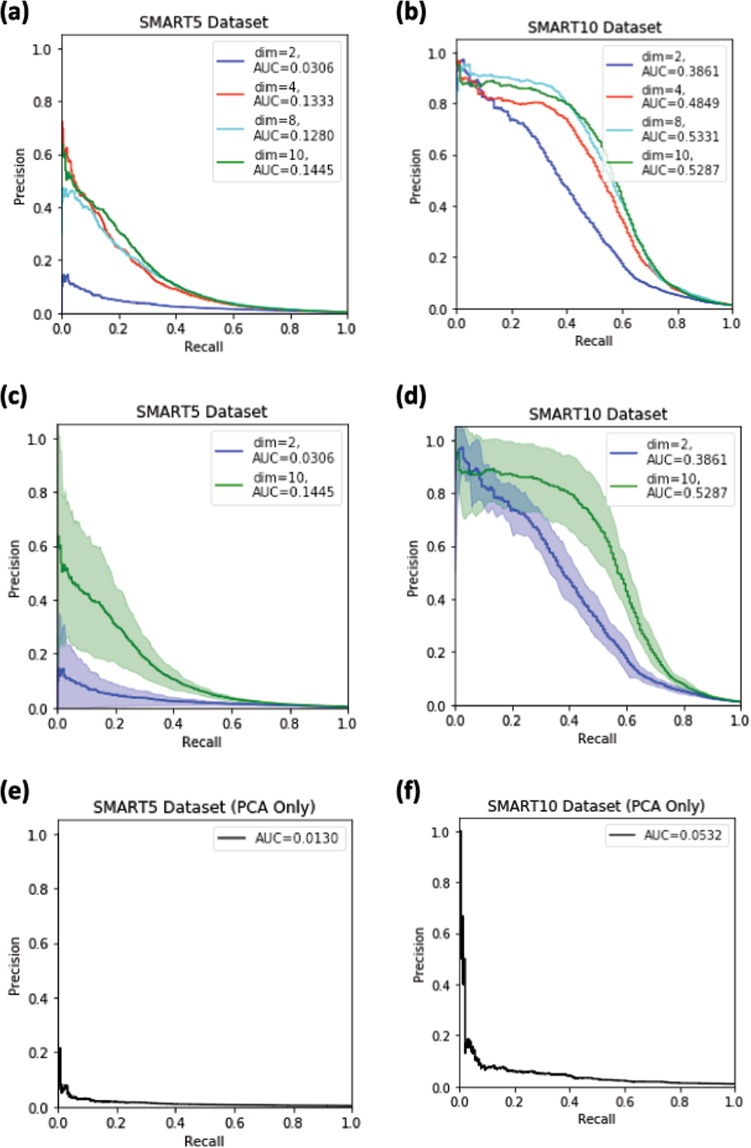
.
