# Supplementary material for: Small Molecule Accurate Recognition Technology (SMART) to Enhance Natural Products Research
Source: Sci Rep. 2017 Oct 27;7:14243. doi: 10.1038/s41598-017-13923-x (PMC5660213; doi:10.1038/s41598-017-13923-x)

**Small Molecule Accurate Recognition Technology (SMART) to Enhance Natural Products Research (Supplementary Information)**

Chen Zhang^1,+^, Yerlan Idelbayev^2,+^, Nicholas Roberts^2^, Yiwen Tao^3,4^, Yashwanth Nannapaneni^2^, Brendan M. Duggan^5^, Jie Min^6^, Eugene C. Lin^7,8^, Erik C. Gerwick^9^, Garrison W. Cottrell^2,*^, and William H. Gerwick^3,5,*^

^1^Department of Nanoengineering, University of California, San Diego, La Jolla, California 92093, United States of America

^2^Department of Computer Science and Engineering, University of California, San Diego, La Jolla, California 92093, United States of America

^3^Center for Marine Biotechnology and Biomedicine, Scripps Institution of Oceanography, La Jolla, California 92037, United States of America

^4^School of Pharmaceutical Sciences, Guangzhou Medical University, Guangzhou, Guangdong 511436, People’s Republic of China

^5^Skaggs School of Pharmacy and Pharmaceutical Sciences, University of California, San Diego, La Jolla, California 92093, United States of America

^6^Department of Electrical and Computer Engineering, University of California, San Diego, La Jolla, California 92093, United States of America

^7^Vanderbilt University Institute of Imaging Science, Vanderbilt University, Nashville, Tennessee, 37235 United States of America

^8^Department of Radiology and Radiological Sciences, Vanderbilt University, Nashville, Tennessee, 37235 United States of America

^9^Physikalisches Institut, Universität Göttingen, Friedrich-Hund-Platz 1, 37077 Göttingen, Germany

[^*^Co-senior and Corresponding Authors: gary@ucsd.edu](mailto:*gary@ucsd.edu); [wgerwick@ucsd.edu](mailto:wgerwick@ucsd.edu)

^+^These authors contributed equally to this work

**Contents of Supplementary Information**

Figure S1. A cluster map containing 400 compounds after 4,800 training iterations 4

Figure S2. A cluster map containing 400 compounds after 4,800 training iterations (Continued) 5

Figure S3. Molecular structures associating with the HSQC spectra within the three green boxes - of Figure 4 in the manuscript 6

Figure S4. An example of preparing input HSQC spectra for SMART 7

Figure S5. ^1^H-^13^C HSQC spectra of viequeamide A2 (2) in CDCl_3_ 8

Figure S6. ^1^H-^13^C HSQC spectra of viequeamide A3 (3) in CDCl_3_ 9

Figure S7. ^1^H-^13^C HSQC spectra of viequeamide B (4) in CDCl_3_ 10

Figure S8. ^1^H-^13^C HSQC spectra of viequeamide C (5) in CDCl_3_ 11

Figure S9. ^1^H-^13^C HSQC spectra of viequeamide D (6) in CDCl_3_ 12

Figure S10. The cluster map containing 400 compounds of the pilot study 13

Figure S11. The cluster map containing 2,054 compounds 14

Figure S12. The cluster map containing 2,054 compounds with labels 15

Table S1. Detailed information regarding the compounds in the blue box of Figure 4 3

Table S2. Top 20 closest compound families for each spectra within the ebractenoids family 16

Table S3. Top 20 closest compound families for each spectra within the naphthomycins family 19

Table S4. Top 20 closest compound families for each spectra within the veraguamides family  20

Table S5. Top 50 closest compound families for each spectra within the viequeamides family   22

References 25

Visualization of 10D embeddings 26

Total number of pairs of inputs in the training process 27

Reasons for software and parameters selection 28

SMART training speed 29

Noisy HSQC spectra of ebractenoid C 30

Noisy HSQC spectra of hyphenrone I 33

Table S1. Detailed information regarding the compounds in the blue box of Figure 4.

| **Molecular Labels** | **Collection Information** | **Bioactivity** |
| --- | --- | --- |
| roots of *Clausena lansium* derived compounds^1^ | collected from Quỳ Hợp District, Nghệ An Province, Vietnam in March 2011 | anti-inflammation, inhibition of superoxide anion generation or elastase release, |
| aaptamine derivatives from the Indonesian sponge *Aaptos suberitoides*^2^ | collected by scuba diving in Ambon, Indonesia in October 1996 at the depth of 3 m | cytotoxic activity against the murine lymphoma L5178Y cell line |
| aaptamine derivatives from the South China Sea Sponge *Aaptos aaptos*^3^ | collected off Woody (Yongxing) Island and Seven Connected Islets in the South China Sea in June 2007 | cytotoxicities against HL60, K562, MCF-7, KB, HepG2, and HT-29 cells |
| alkaloids from the root of *Isatis indigotica*^4^ | collected from Anhui Province, China in December 2009 | antiviral, against influenza virus A/Hanfang/359/95 (H3N2) or inhibition of Coxsackie virus B3 replication |
| casuarinines^5^ | collected from Zhenghe County of Fujian Province, China, in October 2010 | neuroprotective effect against hydrogen peroxide (H_2_O_2_)-induced neuronal cell damage in human neuroblastoma SH-SY5Y cells or inhibition of acetylcholinesterase (AChE) |


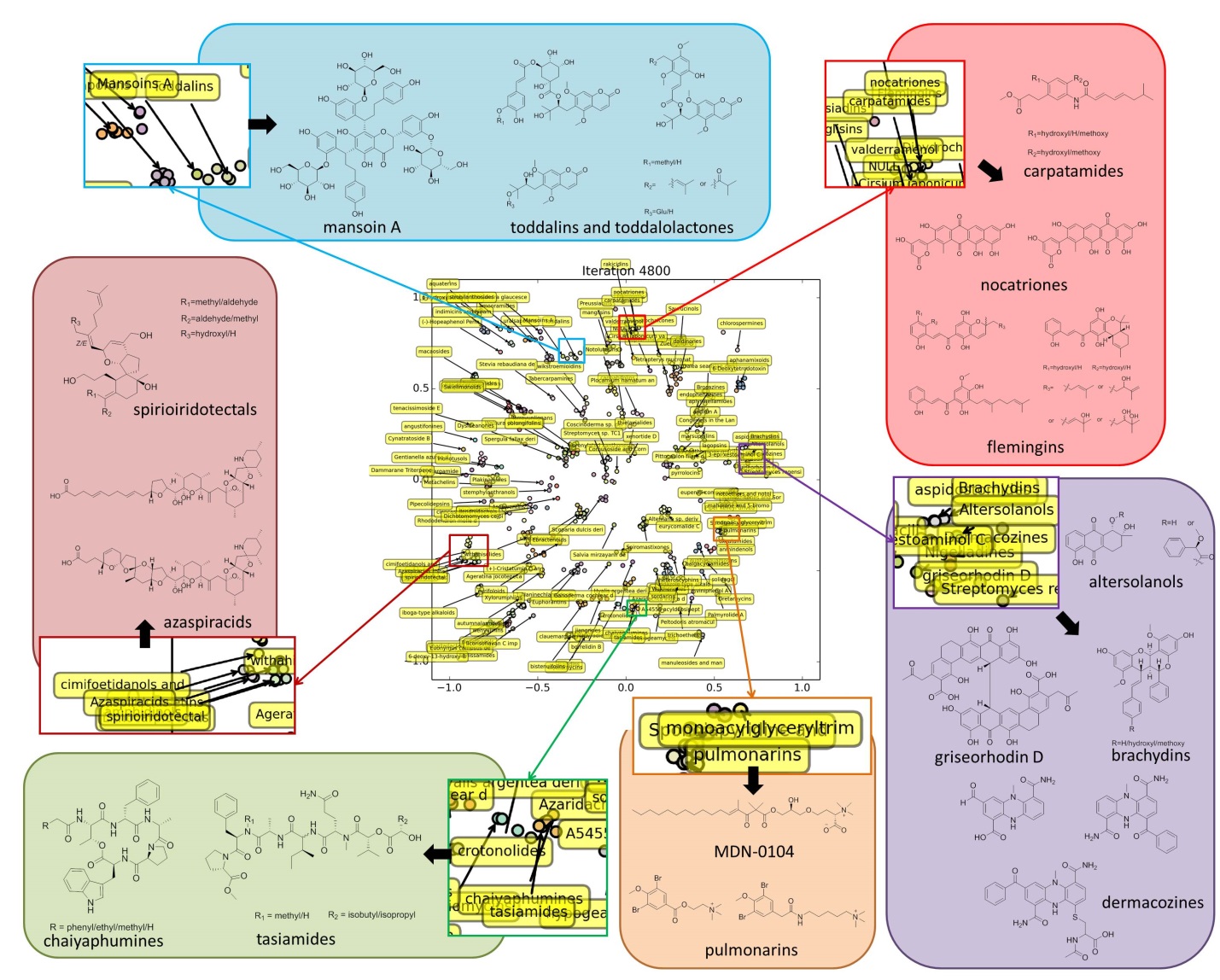


Figure S1. A cluster map containing 400 compounds after 4,800 training iterations. This smaller cluster map shows a distribution of different families of compounds on this map. The names of the compounds are shown as yellow labels.


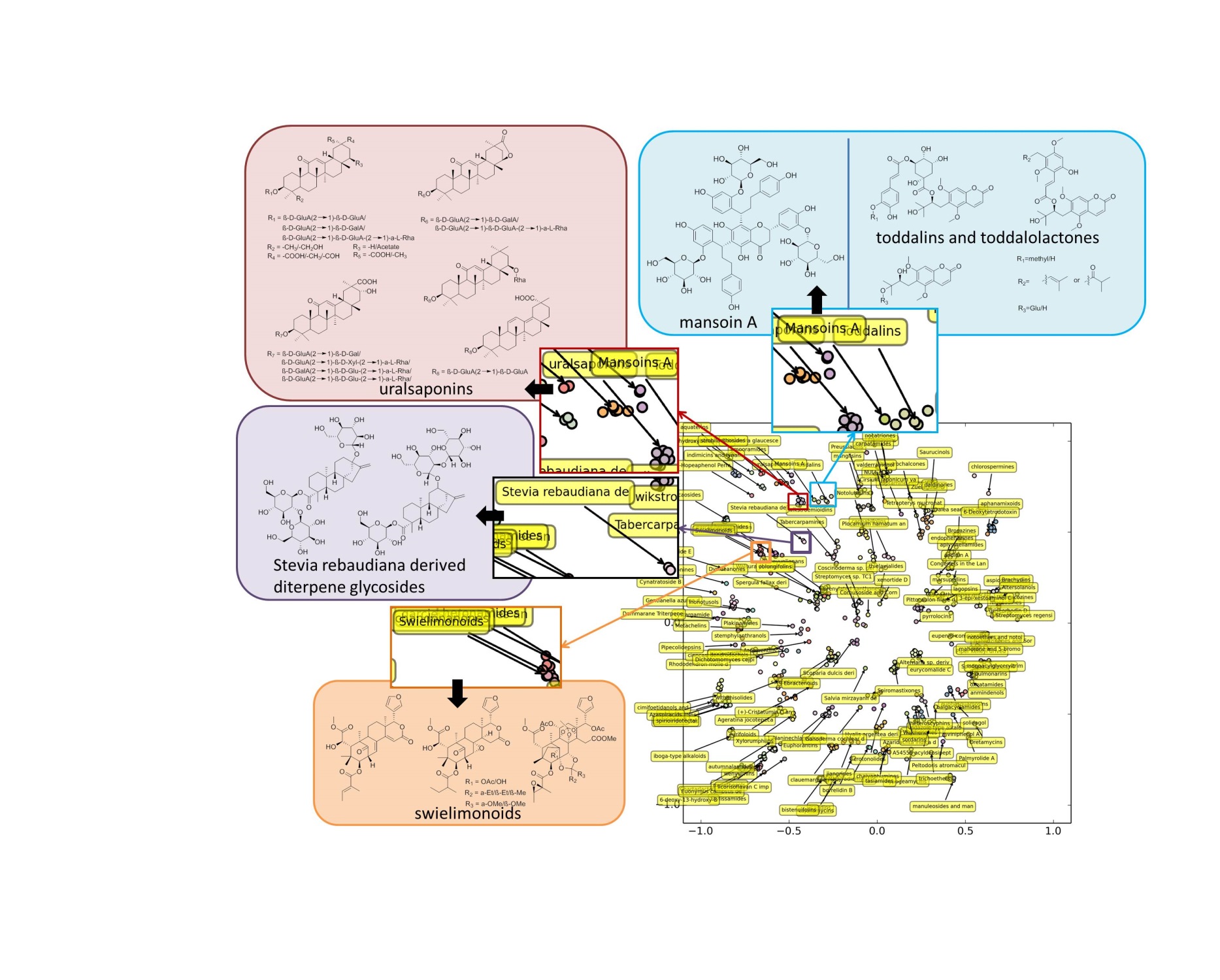


Figure S2. A cluster map containing 400 compounds after 4,800 training iterations. (Continued) The structural change from one family to another appears to be continuous and evolving. Specifically, we observed some intermediates *i.e.* uralsaponins^6^ and *Stevia rebaudiana* derived diterpene glycosides^7^ (saponins) between mansion A^8^ (glycosides) and swielimonoids^9^ (terpenoids).

**
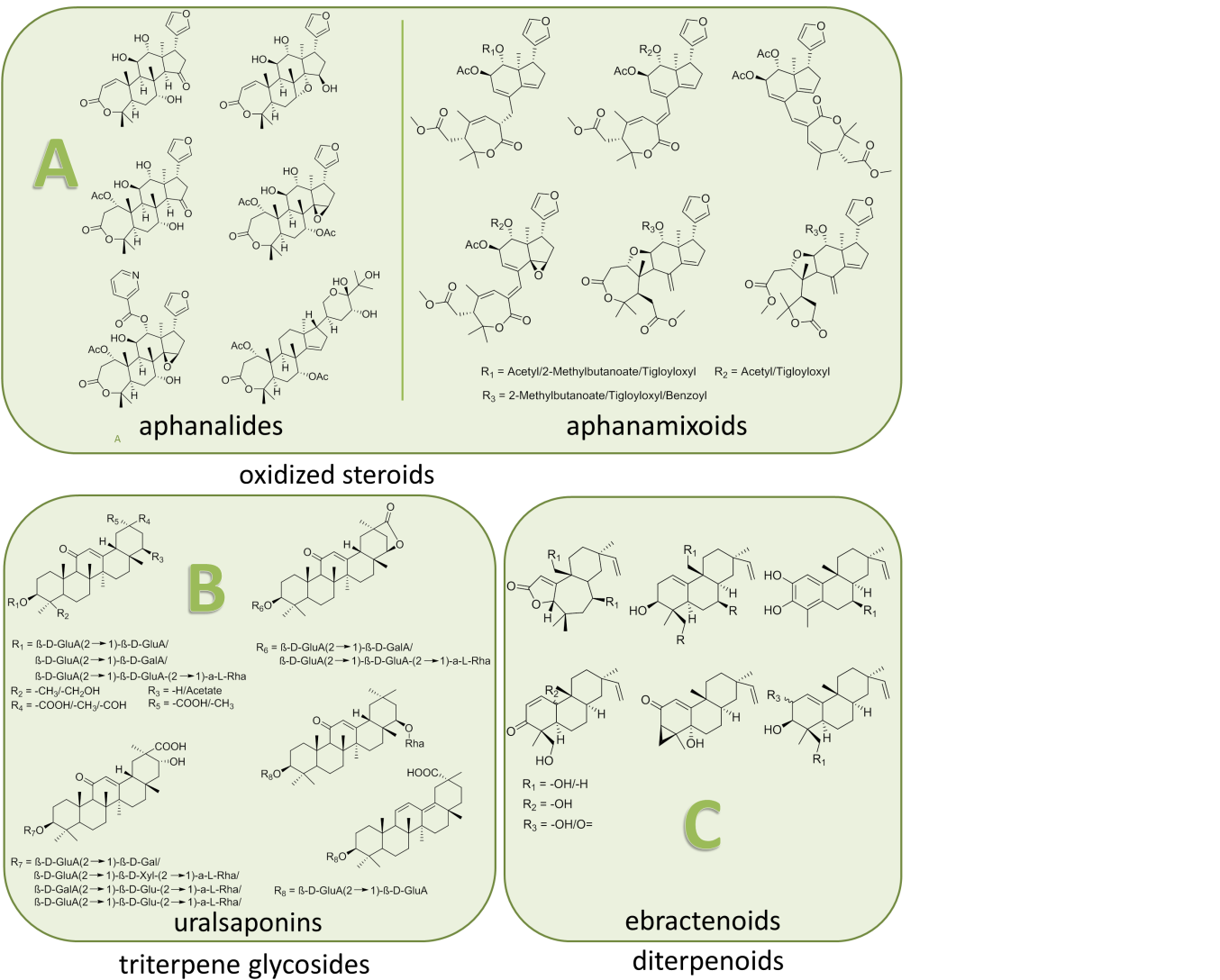
**

Figure S3. Molecular structures associating with the HSQC spectra within the three green boxes of Figure 4 in the manuscript. Cluster A: oxidized steroids from two articles in the Journal of Natural Products, from plants *Aphanamixis polystachya* and *Aphanamixis grandifolia*, respectively. Cluster B: triterpene glycosides isolated from the roots of *Glycyrrhiza uralensis Fisch*. Cluster C: diterpenoids isolated from the roots of *Euphorbia ebracteolata*.


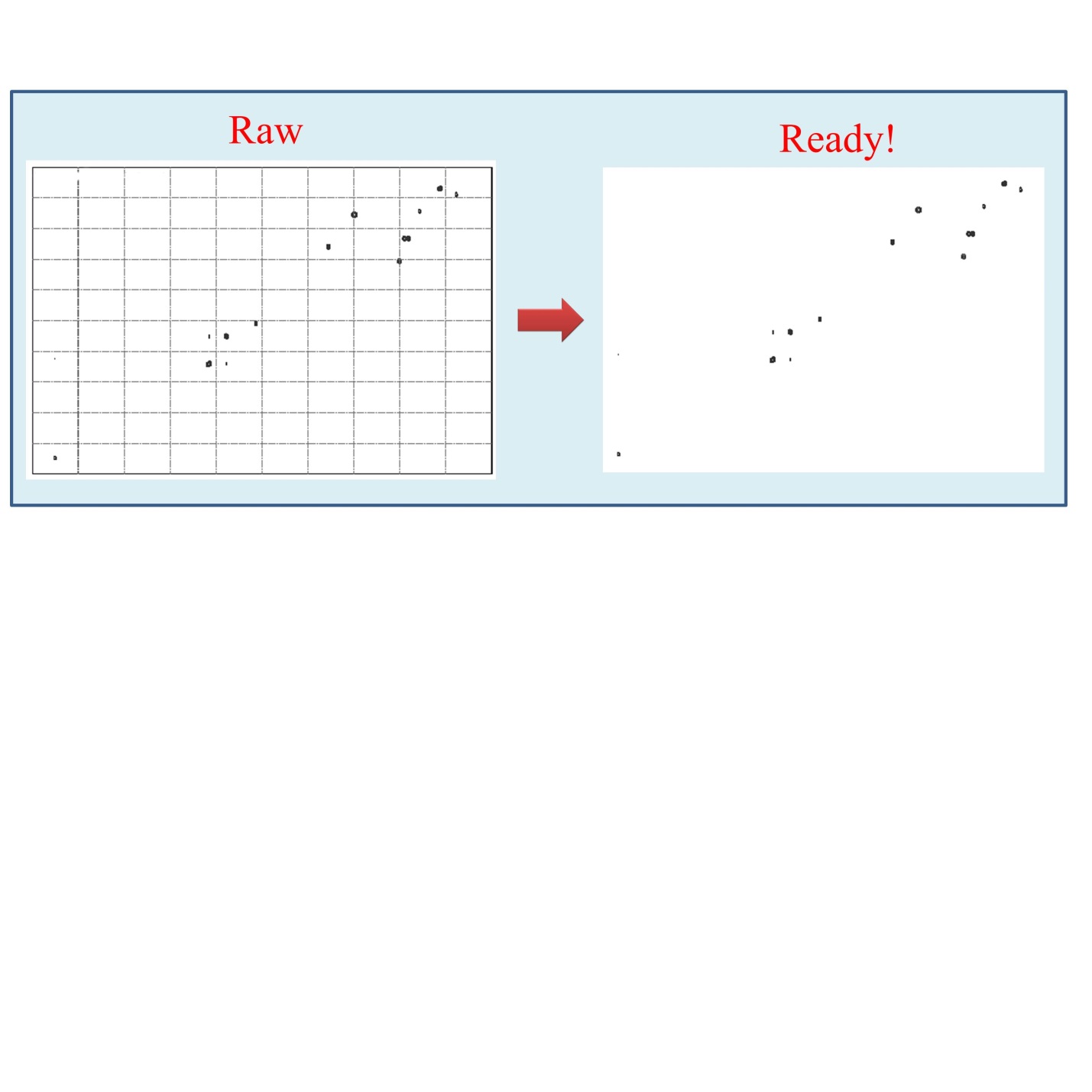


Figure S4. An example of preparing input HSQC spectra for SMART. Adobe Acrobat was used to convert .PDF spectra into .PNG files. Then Adobe Photoshop was used to remove axis labels, frames, grids and artificial marks, if there are any, so that only the HSQC signal patterns and experimental noise were left on a white background. Another important application of Adobe Photoshop in this preparation was to turn the spectra into black (signal and noise) and white (background). Spectra were flipped and rotated if there was a switch between f1 and f2 dimensions to make sure that ^1^H NMR was on the horizontal dimension and ^13^C NMR was on the vertical dimension. Hence, the input of the SMART is a binary image.

**
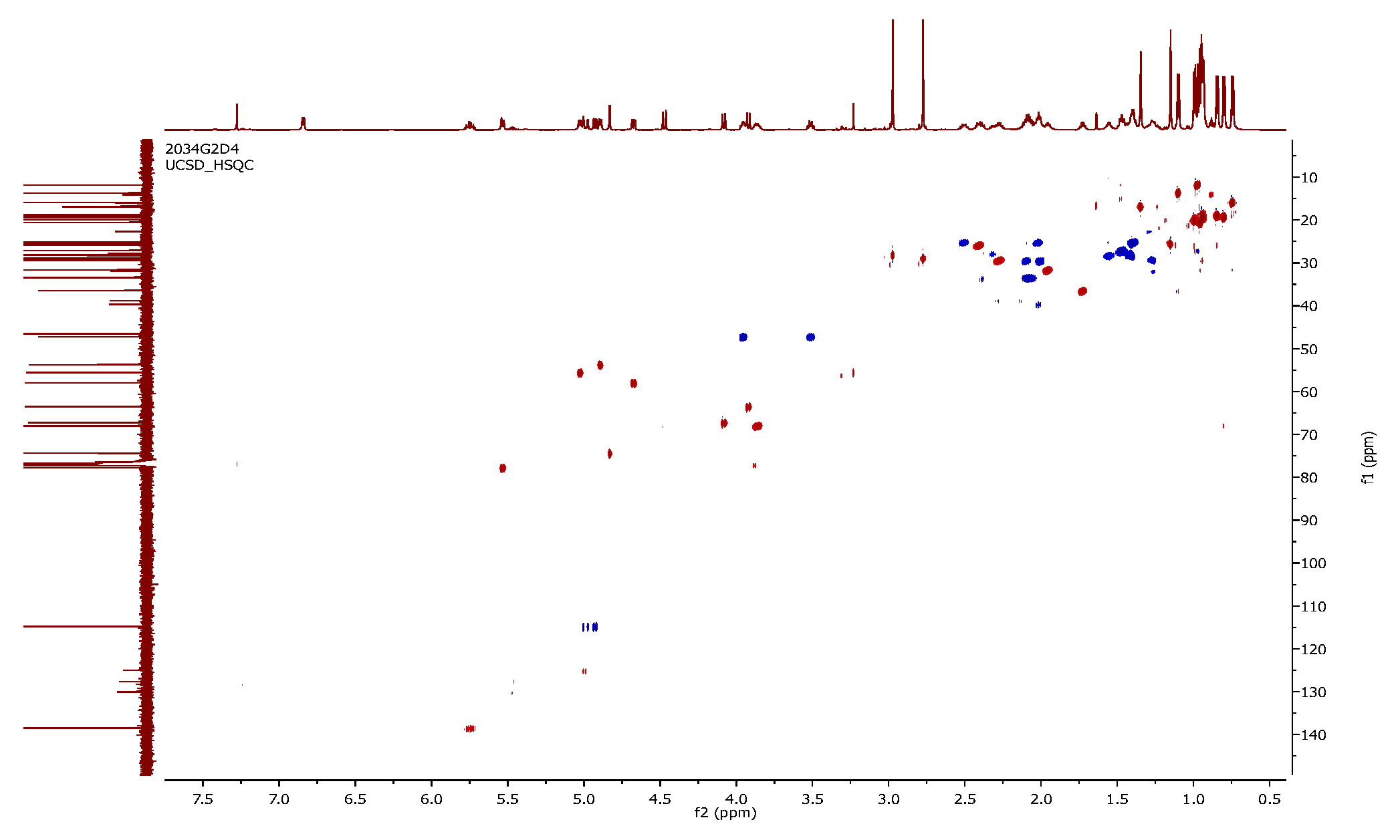
**

Figure S5. ^1^H-^13^C HSQC spectra of viequeamide A2 (2) in CDCl_3_.


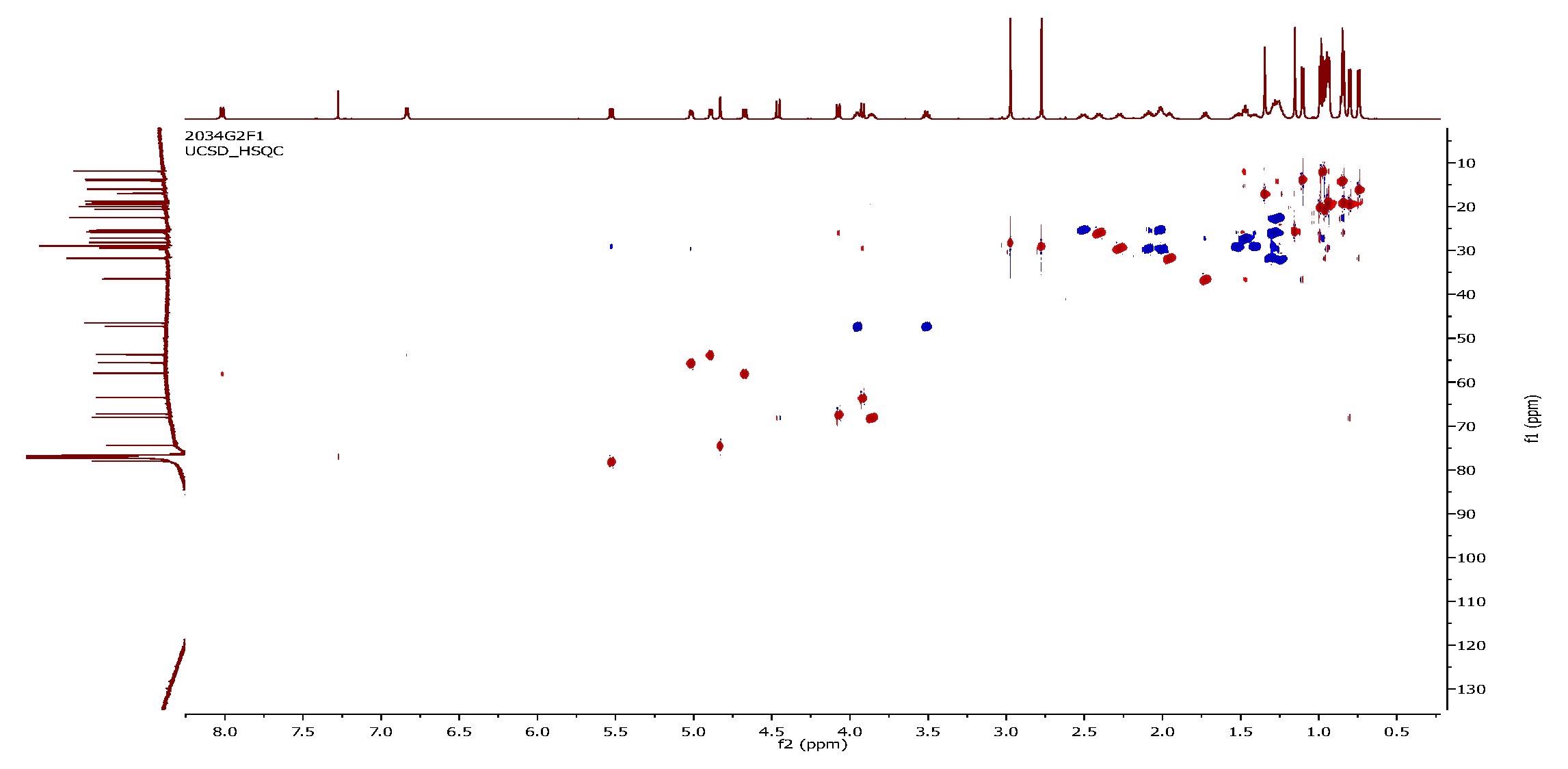


Figure S6. ^1^H-^13^C HSQC spectra of viequeamide A3 (3) in CDCl_3_.

**
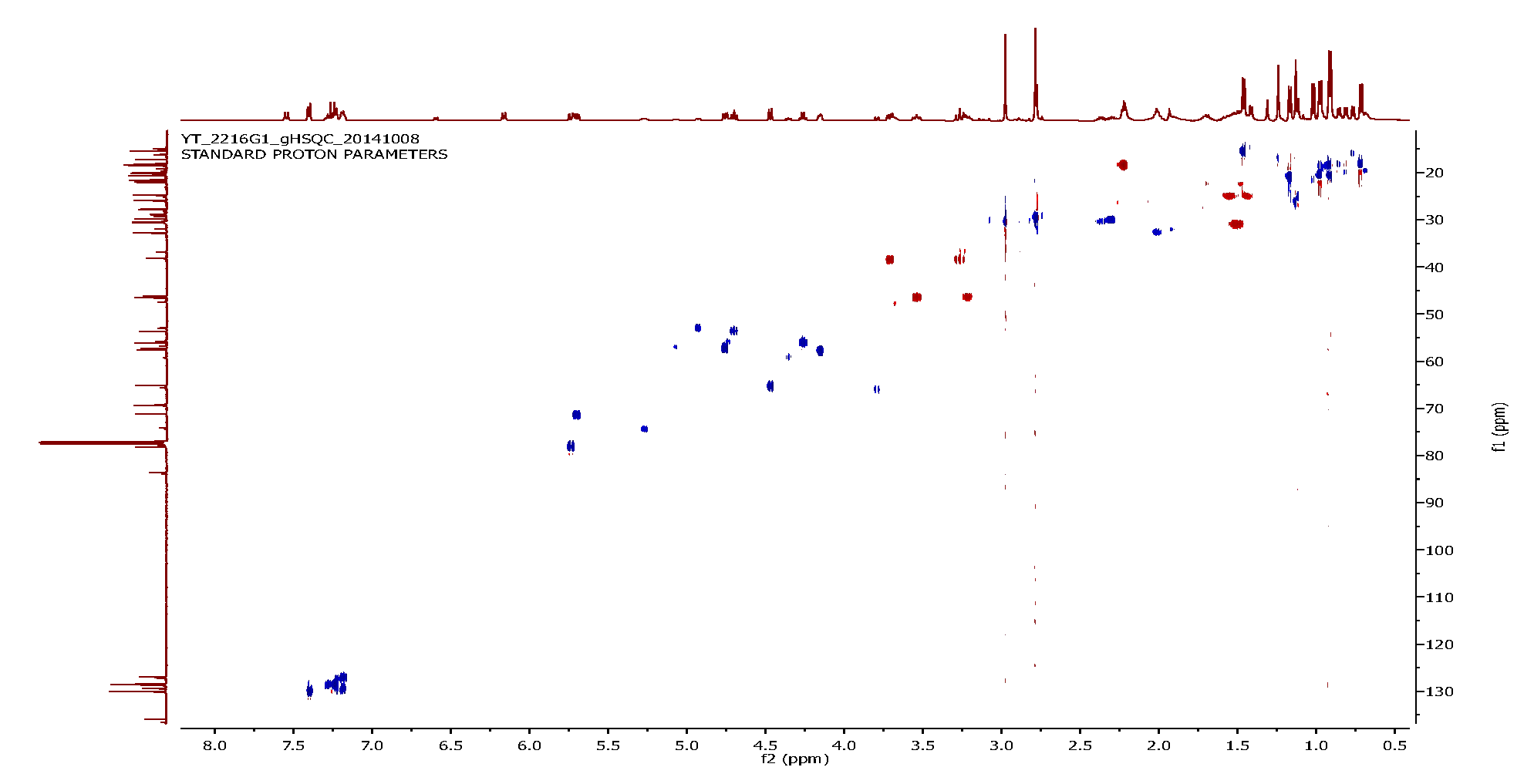
**

**Figure S7. ^1^H-^13^C HSQC spectra of viequeamide B (4) in CDCl_3_.**

**
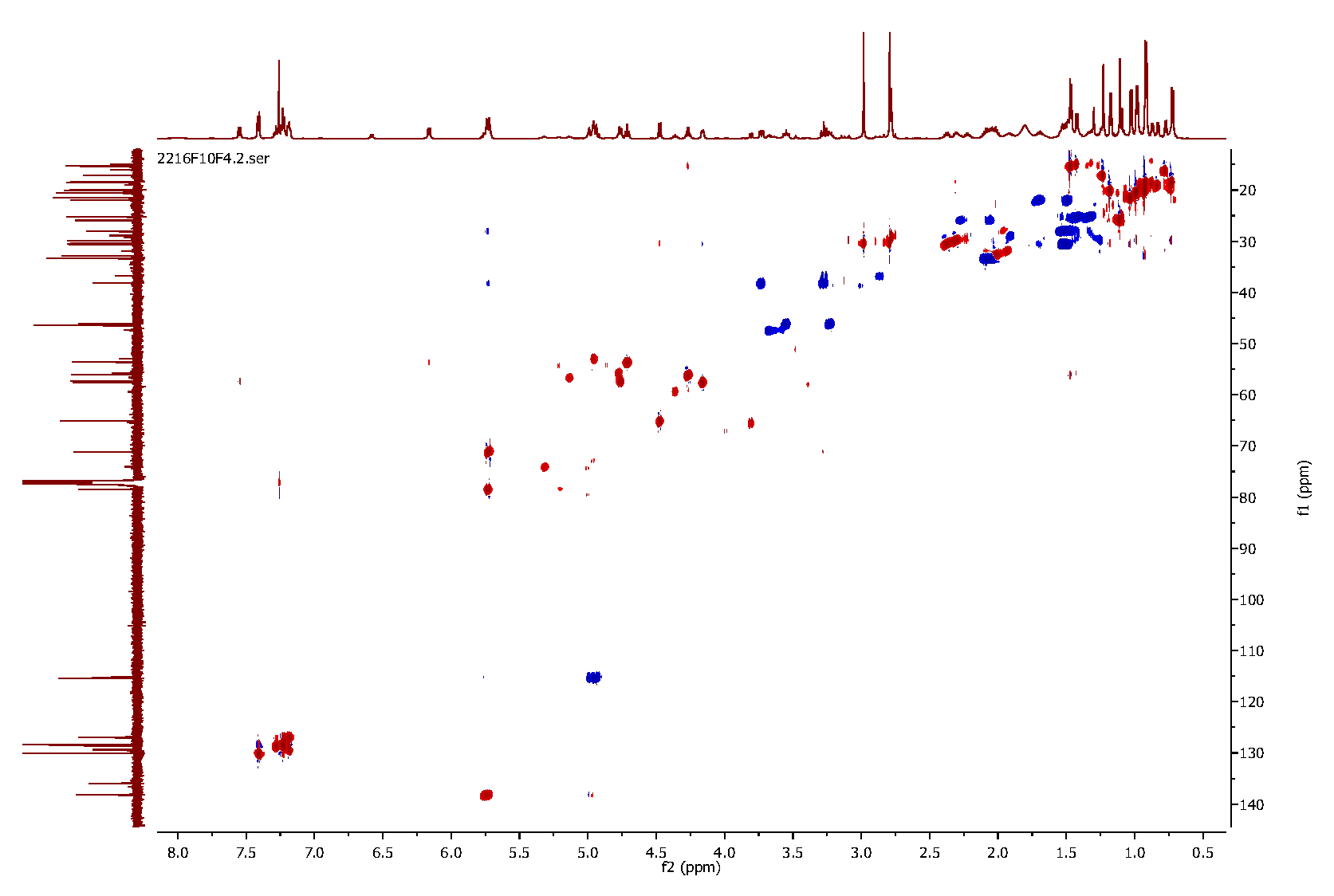
**

**Figure S8. ^1^H-^13^C HSQC spectra of viequeamide C (5) in CDCl_3_.**

**
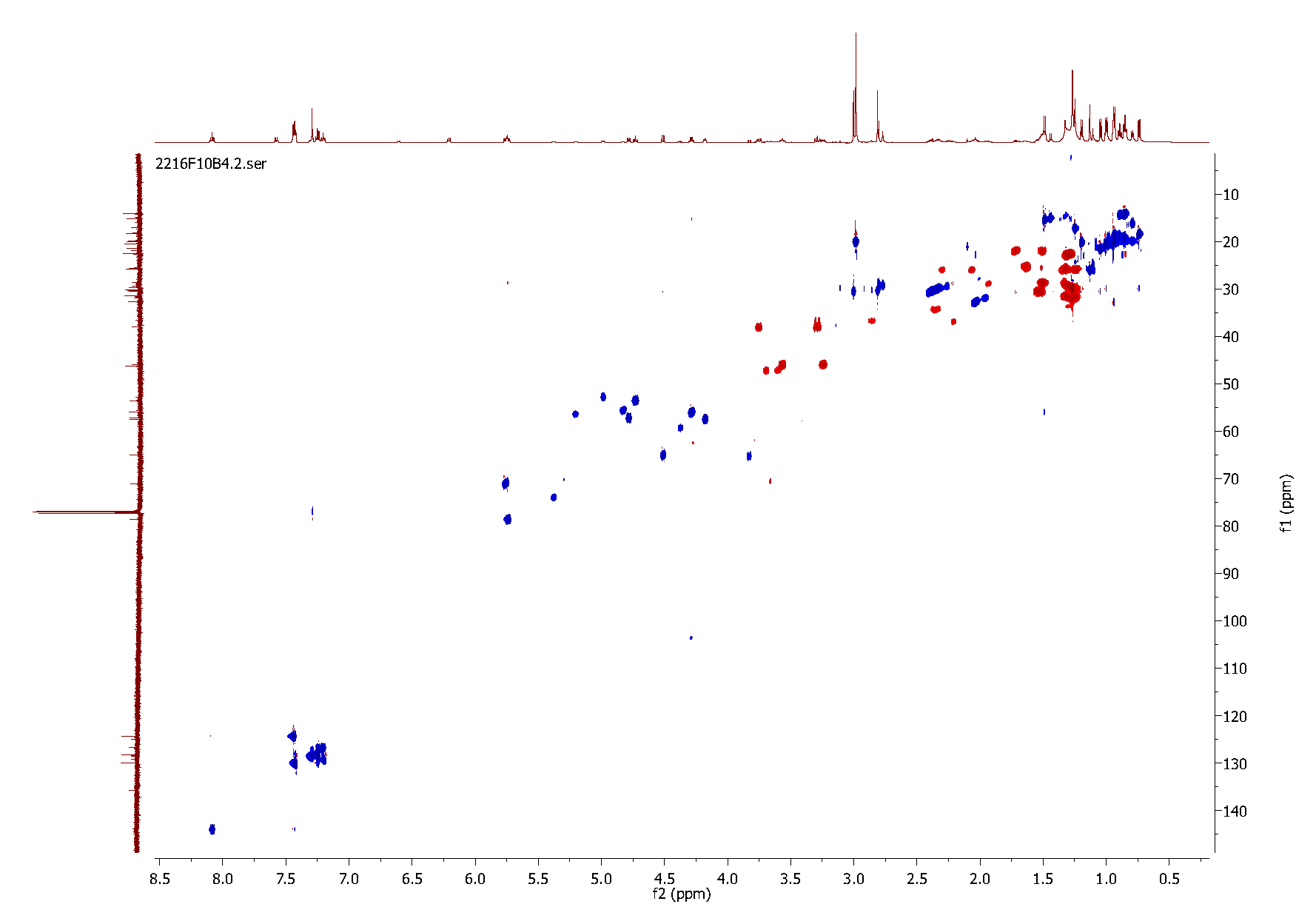
**

**Figure S9. ^1^H-^13^C HSQC spectra of viequeamide D (6) in CDCl_3_.**

**
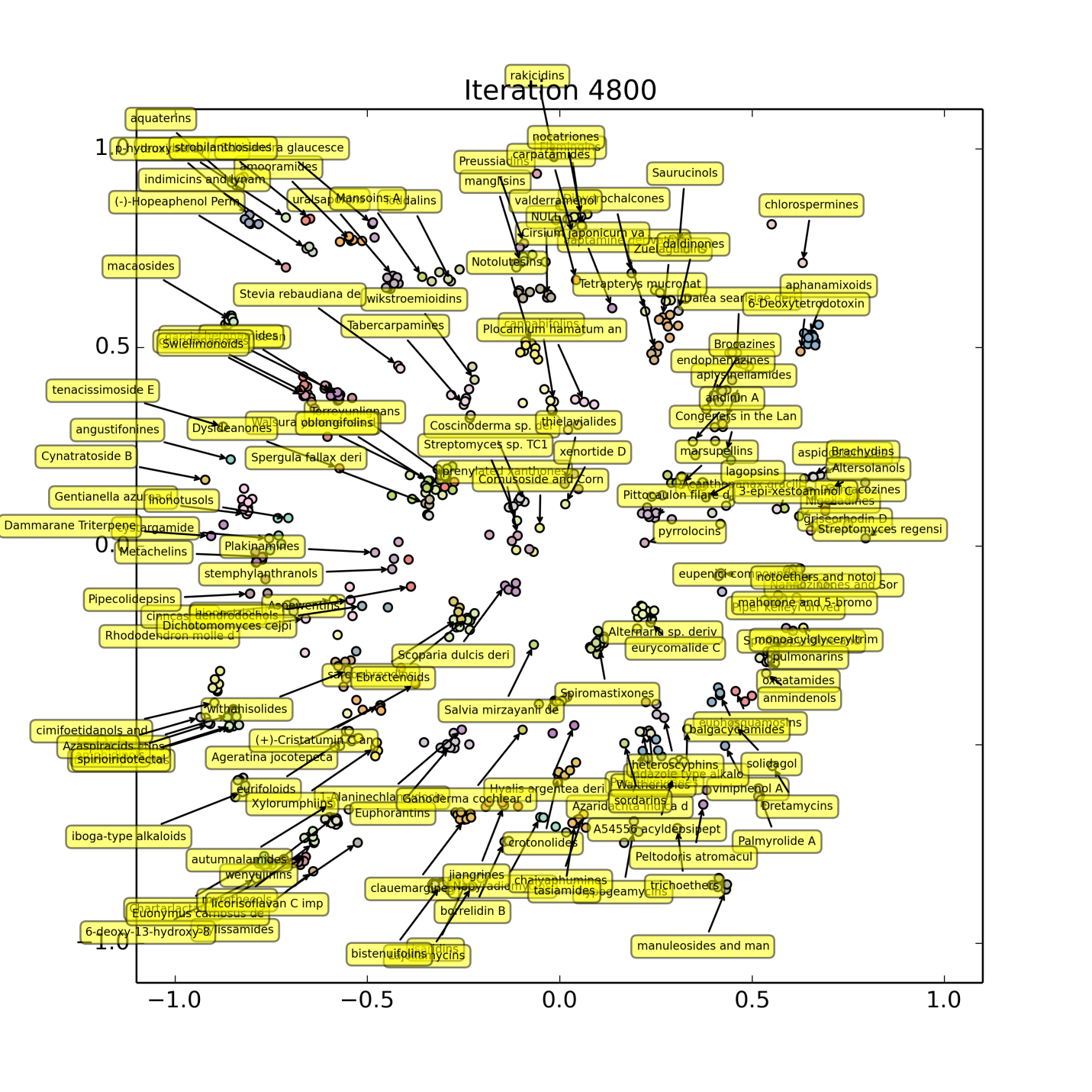
**

**Figure S10. The cluster map containing 400 compounds after 4,800 iterations with node labels.**

**
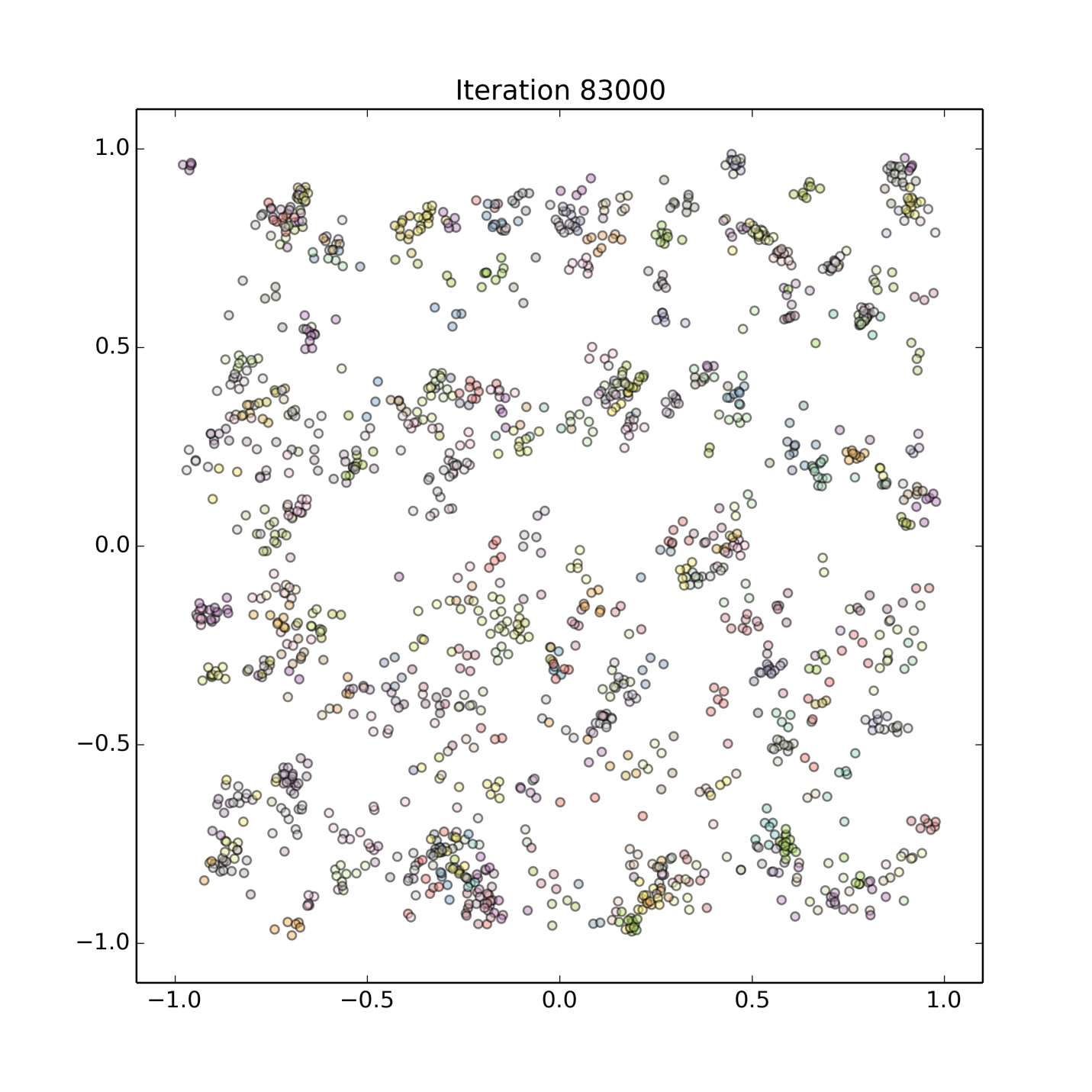
**

**Figure S11. The cluster map containing 2,054 compounds after 83,000 iterations.**

**
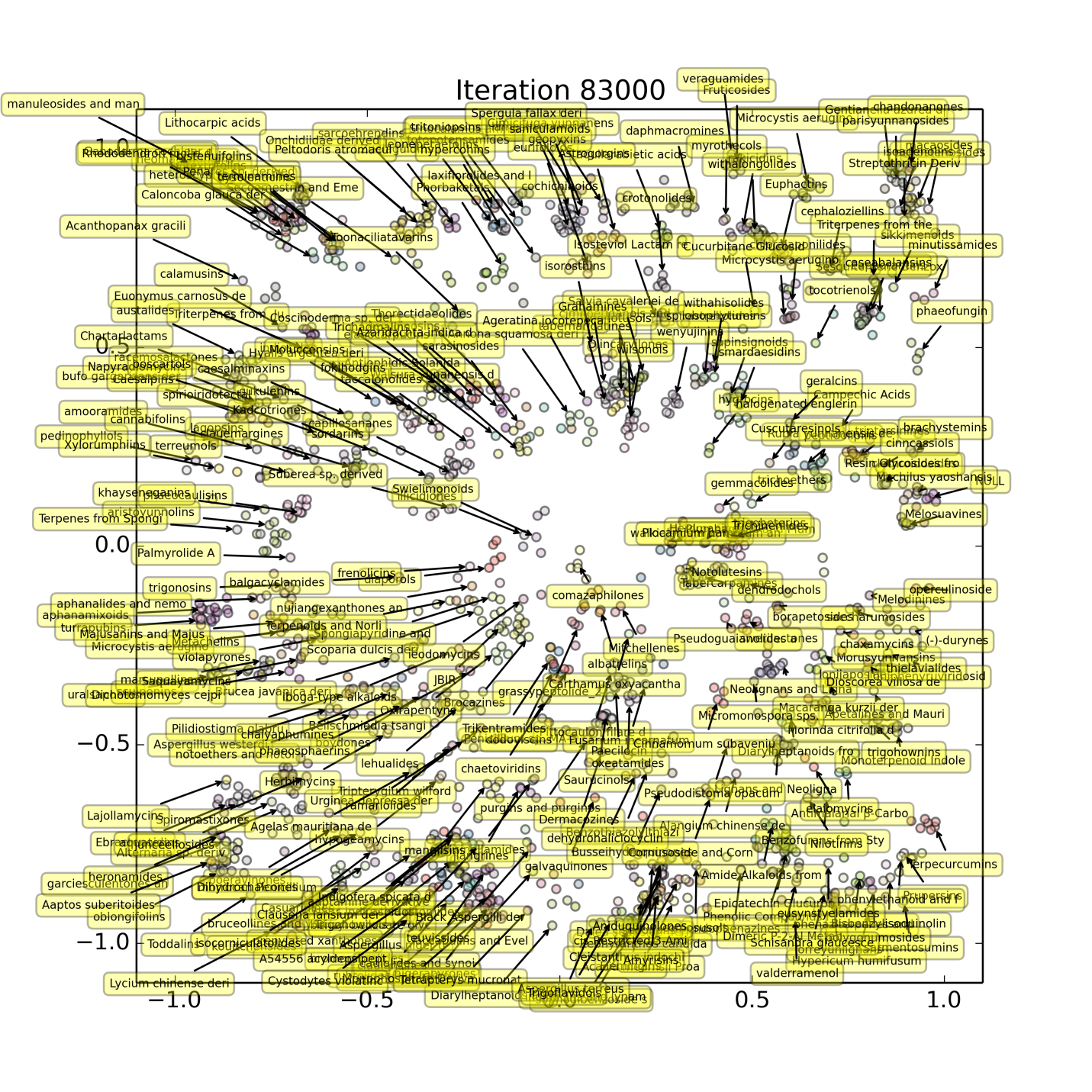
**

**Figure S12. The cluster map containing 2,054 compounds after 83,000 iterations with labels.**

Table S2. Lists of top 20 closest compound families for each spectra within the held-out set of ebractenoids in order of their distance to the test compounds in 10D space.^a^

| **Ebractenoid A** | **Ebractenoid B** | **Ebractenoid C** | **Ebractenoid D** | **Ebractenoid E** | **Ebractenoid F** | **Ebractenoid G** | **Ebractenoid H** | **Ebractenoid I** | **Ebractenoid J** |
| --- | --- | --- | --- | --- | --- | --- | --- | --- | --- |
| bistenuifolins | lamesticumins | diaporols | oblongifolins | uralsaponins | elaeocarpucins | Lignans and Neolignans from Sinocalamus affinis | chaetoviridins | turrapubins | garciesculentones and garciesculenxanthone |
| withalongolides | Onchidiidae derived Bis-γ-pyrone Polypropionates | Brocazines | bistenuifolins | boscartols | khayseneganins | tritoniopsins | Flemingins | turrapubins | amooramides |
| triptersinines | comazaphilones | phaeocaulisins | spirioiridotectal | aquaterins | Saquayamycins | junceellosides | triptersinines | Pittocaulon filare derived sespquiterpenes | Grahamines |
| clauemargines | neomaclafungins | Neolignans and Lignans from Machilus robusta | trigohownins | turrapubins | Acacia mearnsii Proanthocyanidinss | trigonosins | kouitchensides | aphanamixoids | Phorbaketals |
| Astrogorgins | Diarylheptanoids from Alpinia katsumadai | indiosides | saniculamoids | spirioiridotectal | Saquayamycins | triptersinines | Clausena lansium derived compounds | Astrogorgins | amooramides |
| manglisins | gemmacolides | Saquayamycins | spirioiridotectal | Salvia cavaleriei derived ent-Kaurane | macaosides | capillosananes | withahisolides | clauemargines | comazaphilones |
| bistenuifolins | Bisbenzylisoquinoline Alkaloids Stephania epigaea derived | Cimicifuga yunnanensis derived Triterpenes | bruceollines and yadanziolides | notoethers and notoincisols | trigohownins | JBIR-21 | oblongifolins | Astrogorgins | aphanamixoids |
| Astrogorgins | caseabalansins | Diarylheptanoids and Flavonoids from Viscum album | trigonosins | uralsaponins | teuvissides | benzyl benzoate glycosides | Lignans and Neolignans from Sinocalamus affinis | triptersinines | Lithocarpic acids |
| Grahamines | aphanamixoids | Machilus yaoshansis derived dammarane glycosides | calamusins | notoethers and notoincisols | macaosides | macaosides | Sinocalamus affinis derived triterpenoids and steroids | Astrogorgins | Zuelaguidins |
| Chartarlactams | notoethers and notoincisols | Lignans and Neolignans from Sinocalamus affinis | cochichinoids | fokihodgins | Lignans and Neolignans from Sinocalamus affinis | Chartarlactams | teuvissides | Grahamines | Trichagmalins |
| turrapubins | Ganoderma cochlear derived Triterpenoids | clethroidosides | turrapubins | hypogeamycins | Euonymus carnosus derived Lupane Triterpenoids Euonymus carnosus | Pilidiostigma glabrum derived dibenzofurans | Pilidiostigma glabrum derived dibenzofurans | aphanamixoids | Melosuavines |
| rakicidins | Euphorantins | Lycium chinense derived neolignanamides  and lignanamides | Isatis indigotica derived alkaloids | trigonosins | Prupersins | Metachelins | Aniduquinolones | hygrocins | Astrogorgins |
| manglisins | khayseneganins | Rubia yunnanensis derived Arborinane-type Triterpenoids and Anthraquinones | bruceollines and yadanziolides | Neolignans and Lignans from Machilus robusta | trigohownins | Secoemestrin and Emericellenes | Metachelins | Microcystis aeruginosa derived protease inhibitors | terminamines |
| myrothecols | Diarylheptanoids from Alpinia katsumadai | frenolicins | turrapubins | Salvia cavaleriei derived ent-Kaurane | macaosides | khayseneganins | capillosananes | Notolutesins | Benzothiazolylthiazinodihydroisoquinolines from human red hair |
| benzyl benzoate glycosides | Plumbagines and Plumbagosides | taccalonolides | Lignans and Neolignans from Sinocalamus affinis | trigoheterins | Euphactins | aquaterins | Salvia cavaleriei derived ent-Kaurane | Walsura yunanensis derived limonoids | cimifoetidanols and cimifoetidanosides |
| iboga-type alkaloids | Euphorantins | uralsaponins | iboga-type alkaloids | Pittocaulon filare derived sespquiterpenes | benzyl benzoate glycosides | diaporols | diaporols | Chartarlactams | Microcystis aeruginosa derived micropeptins |
| turrapubins | cimifoetidanols and cimifoetidanosides | Machilus yaoshansis derived dammarane glycosides | Saquayamycins | myrothecols | elaeocarpucins | peptidolipins | tritoniopsins | Herdmanines | gemmacolides |
| Diarylheptanoids from Alpinia katsumadai | Astrogorgins | phaeofungin | aristoyunnolins | withalongolides | uralsaponins | notoethers and notoincisols | Alternaria sp. derived metabolites | bistenuifolins | amooramides |
| Walsura yunanensis derived limonoids | Pseudoguaianolides and Guaianolides from Inula hupehensis | Machilus yaoshansis derived dammarane glycosides | trigohownins | teuvissides | triptersinines | Spergula fallax derived glycosides | junceellosides | Swielimonoids | aphanamixoids |
| comazaphilones | indimicins and lynamicins | uralsaponins | Onchidiidae derived Bis-γ-pyrone Polypropionates | Euonymus carnosus derived Lupane Triterpenoids Euonymus carnosus | trigoheterins | Clausena lansium derived compounds | hypogeamycins | aquaterins | aphanalides and nemoralisins |

a. The first row contains the names of each test compound within the ebractenoids family (bold). Compound families that are terpenoids/terpenes within the top 20 closest compounds are coloured red, and can be considered “hits”; non-terpenoids are coloured black. The blocks containing the same family of compounds within the top 20 hits for each ebractenoid are similarly coloured.

Table S3. Lists of top 20 closest compound families for each spectra within the held-out set of naphthomycins in order of their distance to the test compounds in 10D space.^a^

| **naphthomycin L** | **naphthomycin M** | **naphthomycin N** |
| --- | --- | --- |
| Clausena lansium derived compounds | JBIR-94-125 | dysolenticins |
| Euphorantins | rakicidins | Trigohowilols |
| Clausena lansium derived compounds | Pittocaulon filare derived sespquiterpenes | Acacia mearnsii Proanthocyanidinss |
| Trigohowilols | Dimeric P-2-AI Metabolites | Busseihydroquinones |
| Penares sp. derived triterpenoids | laxiflorolides and laxiflorins | Melodinines |
| Triterpenes from the Leaves of Rosa laevigata | Plocamium hamatum and Plocamium costatum derived halogenated monoterpenes | Apetalines and Mauritines |
| Trigohowilols | Morusyunnansins | tritoniopsins |
| albatrelins | Grahamines | trigonosins |
| albatrelins | Annona squamosa derived bistetrahydrofuran annonaceous acetogenines with mosher | Clausena lansium derived compounds |
| triptersinines | Ganoderma cochlear derived Triterpenoids | lonijaposides |
| Onchidiidae derived Bis-γ-pyrone Polypropionates | Ganoderma cochlear derived Triterpenoids | Ganoderma cochlear derived Triterpenoids |
| Notolutesins | neomaclafungins | Triterpenes from the Leaves of Rosa laevigata |
| Neolignans and Lignans from Machilus robusta | diaporols | sapinsignoids |
| withahisolides | aquilarabietic acids | Euphorantins |
| Terpecurcumins | Monoterpenoid Indole Alkaloids from Gardneria ovata | Herdmanines |
| Oxirapentyns | aquilarabietic acids | comazaphilones |
| comazaphilones | Euphorantins | Cimicifuga yunnanensis derived Triterpenes |
| Amide Alkaloids from Piper boehmeriaefolium | Melosuavines | withahisolides |
| Zephyranthes candida derived alkaloids | withalongolides | cephaloziellins |
| Cornusoside and Cornolactones | dodoviscins | Notolutesins |

a. The first row contains the names of each test compound within the naphthomycins family (bold). Compound families that are polyketides within the top 20 closest compounds are coloured red, and can be considered “hits”; non-polyketides are coloured black. The blocks containing the same family of compounds within the top 20 hits for each naphthomycin are similarly coloured.

Table S4. Lists of top 20 closest compound families for each spectra within the held-out set of veraguamides in order of their distance to the test compounds in 10D space.^a^

| **tetrahydroveraguamide A** | **veraguamide A** | **veraguamide B** | **veraguamide C** | **veraguamide D** | **veraguamide E** | **veraguamide F** | **veraguamide G** |
| --- | --- | --- | --- | --- | --- | --- | --- |
| autumnalamides | noroleanane triterpenoids from Paeonia rockii | austalides | Lithocarpic acids | Lithocarpic acids | Microcystis aeruginosa derived protease inhibitors | austalides | withalongolides |
| terminamines | autumnalamides | Microcystis aeruginosa derived protease inhibitors | minutissamides | grassypeptolides | Tauromantellic acid | Aeruginosins | noroleanane triterpenoids from Paeonia rockii |
| phoslactomycins | Caesalpins | noroleanane triterpenoids from Paeonia rockii | lamesticumins | Peltodoris atromaculata derived fulvinols | austalides | Tauromantellic acid | Lithocarpic acids |
| triptersinines | wilsonols | Siderophore from Streptomyces sp. YM5-799 | geldanamycins | geldanamycins | Melosuavines | Balticidins | cimifoetidanols and cimifoetidanosides |
| phaeocaulisins | Moluccensins | cimifoetidanols and cimifoetidanosides | grassypeptolides | lamesticumins | Lithocarpic acids | Lithocarpic acids | Lithocarpic acids |
| wilsonols | junceellosides | withalongolides | sarcoehrendins | Onchidiidae derived Bis-γ-pyrone Polypropionates | NN′-Methyleno-didemnin A from Trididemnum solidum | Melosuavines | Ganoderma cochlear derived Triterpenoids |
| lystabactins | lagopsins | Plocamium hamatum and Plocamium costatum derived halogenated monoterpenes | NN′-Methyleno-didemnin A from Trididemnum solidum | Lithocarpic acids | Aeruginosins | Microcystis aeruginosa derived protease inhibitors | cochichinoids |
| cochichinoids | racemosalactones | Walsura yunanensis derived limonoids | lamesticumins | NN′-Methyleno-didemnin A from Trididemnum solidum | Siderophore from Streptomyces sp. YM5-799 | Walsura yunanensis derived limonoids | spirastrellolides |
| noroleanane triterpenoids from Paeonia rockii | Penicillium sp. MA-37 derived meroterpenoids and diphenyl ethers | cimifoetidanols and cimifoetidanosides | Lithocarpic acids | minutissamides | noroleanane triterpenoids from Paeonia rockii | eusynstyelamides | terminamines |
| caesalminaxins | dysolenticins | Lithocarpic acids | Microcystis aeruginosa derived protease inhibitors | Phorbaketals | cimifoetidanols and cimifoetidanosides | pouosides | viequeamides |
| dysolenticins | sarasinosides | NN′-Methyleno-didemnin A from Trididemnum solidum | noroleanane triterpenoids from Paeonia rockii | noroleanane triterpenoids from Paeonia rockii | Microcystis aeruginosa derived protease inhibitors | Siderophore from Streptomyces sp. YM5-799 | hypercohins |
| austalides | Cedrus deodara derived tubulin inhibitors | Aeruginosins | cimifoetidanols and cimifoetidanosides | cimifoetidanols and cimifoetidanosides | sarcoehrendins | cimifoetidanols and cimifoetidanosides | austalides |
| viequeamides | austalides | taccalonolides | isorosthins | sarcoehrendins | sikkimenoids | noroleanane triterpenoids from Paeonia rockii | cimifoetidanols and cimifoetidanosides |
| sarcoehrendins | viequeamides | borapetosides | Pipecolidepsins | Pittocaulon filare derived sespquiterpenes | Chartarlactams | isorosthins | grassypeptolide_2 |
| sarasinosides | dysolenticins | sarcoehrendins | austalides | Microcystis aeruginosa derived protease inhibitors | Balticidins | cimifoetidanols and cimifoetidanosides | garciesculentones and garciesculenxanthone |
| tasiamides | sarcoehrendins | Lithocarpic acids | Peltodoris atromaculata derived fulvinols | Onchidiidae derived Bis-γ-pyrone Polypropionates | Microcystis aeruginosa derived protease inhibitors | Microcystis aeruginosa derived protease inhibitors | Euphorantins |
| terminamines | gukulenins | Tabercarpamines | Onchidiidae derived Bis-γ-pyrone Polypropionates | noroleanane triterpenoids from Paeonia rockii | grassypeptolides | brachystemins | noroleanane triterpenoids from Paeonia rockii |
| dysolenticins | phoslactomycins | Microcystis aeruginosa derived protease inhibitors | homotemsirolimuses | minutissamides | tasiamides | Tauromantellic acid | cephaloziellins |
| austalides | austalides | Grahamines | Sesquiterpene Benzoxazoles and Sesquiterpene Quinones from Dactylospongia elegans | Sinocalamus affinis derived triterpenoids and steroids | lystabactins | NN′-Methyleno-didemnin A from Trididemnum solidum | cimifoetidanols and cimifoetidanosides |
| dysolenticins | sarasinosides | hypercohins | Onchidiidae derived Bis-γ-pyrone Polypropionates | Lithocarpic acids | Sesquiterpene Benzoxazoles and Sesquiterpene Quinones from Dactylospongia elegans | Microcystis aeruginosa derived protease inhibitors | Onchidiidae derived Bis-γ-pyrone Polypropionates |

a. The first row contains the names of each test compound within the veraguamides family (bold). Compound families that are peptides within the top 20 closest compounds are coloured red, and can be considered “hits”; non-peptides are coloured black. The blocks containing the same family of compounds within the top 20 hits for each veraguamide are similarly coloured.

Table S5. Lists of top 50 closest compound families for each spectra within the newly isolated members of the viequeamides family in order of their distance to the test compounds in 10D space.^a^

| **viequeamide A** | **viequeamide A2** | **viequeamide A3^b^** | **viequeamide B** | **viequeamide C^b^** | **viequeamide D** |
| --- | --- | --- | --- | --- | --- |
|
| cimifoetidanols and cimifoetidanosides | khayseneganins | aphanamixoids | Astrogorgins | cephaloziellins | cimifoetidanols and cimifoetidanosides |
| cephaloziellins | Euphorantins | Swielimonoids | gemmacolides | wenyujinins | sarasinosides |
| sarasinosides | Astrogorgins | tabernaricatines | gemmacolides | myrothecols | Sesquiterpene Benzoxazoles and Sesquiterpene Quinones from Dactylospongia elegans |
| Sesquiterpene Benzoxazoles and Sesquiterpene Quinones from Dactylospongia elegans | turrapubins | wenyujinins | Astrogorgins | Notolutesins | spirastrellolides |
| spirastrellolides | Gentianella azurea derived triterpenoids | Swielimonoids | teuvissides | vitextrifolins | jiangrines |
| jiangrines | Euphorantins | Plocamium hamatum and Plocamium costatum derived halogenated monoterpenes | aphanamixoids | myrothecols | cimifoetidanols and cimifoetidanosides |
| Gentianella azurea derived triterpenoids | aphanamixoids | aphanamixoids | Notolutesins | cimifoetidanols and cimifoetidanosides | cephaloziellins |
| chlorajaponilides | Triterpenes from the Leaves of Rosa laevigata | hypercohins | Grahamines | Gentianella azurea derived triterpenoids | chlorajaponilides |
| Cucurbitane Glucosides from  Machilus yaoshansis | sedonans | Clausena lansium derived compounds | cephaloziellins | caseabalansins | Cucurbitane Glucosides from  Machilus yaoshansis |
| cimifoetidanols and cimifoetidanosides | Swielimonoids | veraguamides | Plocamium hamatum and Plocamium costatum derived halogenated monoterpenes | Sesquiterpene Benzoxazoles and Sesquiterpene Quinones from Dactylospongia elegans | Gentianella azurea derived triterpenoids |
| caseabalansins | gemmacolides | Torreyunlignans | morrocan Penicillium citrinum derived compounds | Astrogorgins | Phorbaketals |
| caseabalansins | Gentianella azurea derived triterpenoids | Aeruginosins | Dimeric P-2-AI Metabolites | aphanamixoids | caseabalansins |
| Phorbaketals | cannabifolins | veraguamides | Ebractenoids | Gentianella azurea derived triterpenoids | caseabalansins |
| Dalea searlsiae derived compounds | withalongolides | Xylorumphiins | gemmacolides | Walsura yunanensis derived limonoids | veraguamides |
| veraguamides | turrapubins | triptersinines | Plumbagines and Plumbagosides | gemmacolides | Dalea searlsiae derived compounds |
| Euphorantins | lamesticumins | spirastrellolides | khayseneganins | Dalea searlsiae derived compounds | lamesticumins |
| uralsaponins | cephaloziellins | cephaloziellins | aquaterins | spirastrellolides | Euphorantins |
| Euphorantins | viequeamides | aphanalides and nemoralisins | Torreyunlignans | cimifoetidanols and cimifoetidanosides | Swielimonoids |
| cimifoetidanols and cimifoetidanosides | Pittocaulon filare derived sespquiterpenes | triptersinines | Walsura yunanensis derived limonoids | Phorbaketals | cimifoetidanols and cimifoetidanosides |
| myrothecols | Microcystis aeruginosa derived protease inhibitors | Scoparia dulcis derived diterpnoids | Pittocaulon filare derived sespquiterpenes | Swielimonoids | Euphorantins |
| Notolutesins | aphanamixoids | Notolutesins | spirastrellolides | laxiflorolides and laxiflorins | Gentianella azurea derived triterpenoids |
| Swielimonoids | uralsaponins | khayseneganins | aphanamixoids | Gentianella azurea derived triterpenoids | Gentianella azurea derived triterpenoids |
| Gentianella azurea derived triterpenoids | Bisbenzylisoquinoline Alkaloids Stephania epigaea derived | Swielimonoids | viequeamides | Acacia mearnsii Proanthocyanidinss | myrothecols |
| lamesticumins | Euphorantins | Herdmanines | amooramides | aphanamixoids | uralsaponins |
| Euphorantins | aphanamixoids | Tripterygium wilfordii derived dihydroagarofurans | lamesticumins | Grahamines | Euphorantins |
| wenyujinins | tritoniopsins | khayseneganins | fokihodgins | Sesquiterpene Benzoxazoles and Sesquiterpene Quinones from Dactylospongia elegans | Notolutesins |
| Gentianella azurea derived triterpenoids | cimifoetidanols and cimifoetidanosides | Plocamium hamatum and Plocamium costatum derived halogenated monoterpenes | Scoparia dulcis derived diterpnoids | aphanamixoids | chandonanones |
| cimifoetidanols and cimifoetidanosides | Dalea searlsiae derived compounds | Trichagmalins | Dalea searlsiae derived compounds | sarasinosides | cephaloziellins |
| viequeamides | bistenuifolins | cimifoetidanols and cimifoetidanosides | cimifoetidanols and cimifoetidanosides | caseabalansins | Pittocaulon filare derived sespquiterpenes |
| Pittocaulon filare derived sespquiterpenes | Brocazines | withahisolides | Caesalpins | Onchidiidae derived Bis-γ-pyrone Polypropionates | cimifoetidanols and cimifoetidanosides |
| morrocan Penicillium citrinum derived compounds | caseabalansins | Grahamines | uralsaponins | Euphorantins | Gentianella azurea derived triterpenoids |
| chandonanones | neomaclafungins | Xylorumphiins | Fruticosides | marsupellins | viequeamides |
| cephaloziellins | Apetalines and Mauritines | Astrogorgins | nujiangexanthones and nujiangefolins | Euphorantins | Oxirapentyns |
| aphanamixoids | chandonanones | Diarylheptanoids from Alpinia katsumadai | uralsaponins | Euphorantins | wenyujinins |
| Gentianella azurea derived triterpenoids | veraguamides | Clausena lansium derived compounds | Swielimonoids | Notolutesins | bistenuifolins |
| Swielimonoids | neomaclafungins | Diarylheptanoids from Alpinia katsumadai | Salvia cavaleriei derived ent-Kaurane | morrocan Penicillium citrinum derived compounds | morrocan Penicillium citrinum derived compounds |
| Oxirapentyns | chlorajaponilides | withalongolides | Xylorumphiins | cimifoetidanols and cimifoetidanosides | tritoniopsins |
| cephaloziellins | jiangrines | cimifoetidanols and cimifoetidanosides | bistenuifolins | Zuelaguidins | Swielimonoids |
| Annona squamosa derived bistetrahydrofuran annonaceous acetogenines with mosher | diaporols | endophenazines | aphanamixoids | Bisbenzylisoquinoline Alkaloids Stephania epigaea derived | cephaloziellins |
| tritoniopsins | veraguamides | aphanamixoids | aphanamixoids | Gentianella azurea derived triterpenoids | aphanamixoids |
| bistenuifolins | cephaloziellins | trigohownins | cimifoetidanols and cimifoetidanosides | Dimeric P-2-AI Metabolites | cephaloziellins |
| aphanamixoids | Cucurbitane Glucosides from  Machilus yaoshansis | gemmacolides | Euphorantins | cephaloziellins | aphanamixoids |
| aphanamixoids | gemmacolides | pedinophyllols | comazaphilones | Walsura yunanensis derived limonoids | Astrogorgins |
| cephaloziellins | Cimicifuga yunnanensis derived Triterpenes | teuvissides | Trichagmalins | Cucurbitane Glucosides from  Machilus yaoshansis | Annona squamosa derived bistetrahydrofuran annonaceous acetogenines with mosher |
| Astrogorgins | Oxirapentyns | withalongolides | aphanalides and nemoralisins | jiangrines | veraguamides |
| veraguamides | turrapubins | Euphactins | aphanamixoids | amooramides | aphanamixoids |
| Grahamines | caesalminaxins | Walsura yunanensis derived limonoids | endophenazines | sarcoehrendins | isorosthins |
| Astrogorgins | bruceollines and yadanziolides | gemmacolides | tabernaricatines | chlorajaponilides | khayseneganins |
| khayseneganins | neomaclafungins | Walsura yunanensis derived limonoids | Zuelaguidins | teuvissides | Astrogorgins |
| Dalea searlsiae derived compounds | isorosthins | cephaloziellins | hypercohins | amooramides | gemmacolides |

a. Close compound families of each newly obtained viequeamide spectra are listed in each column with the compound names in bold. Compounds in the viequeamide family are highlighted in red; non-viequeamides are coloured black. The blocks containing the same peptidic family of compounds within the top 50 hits for each of the viequeamides are similarly coloured.

b. Due to the small number of viequeamides in the training set (only 2), viequeamides A3 and C were not closely associated with this family.

# References:

1 Shen, D. Y. *et al.* Constituents of the Roots of Clausena lansium and Their Potential Anti-inflammatory Activity. *J Nat Prod* **77**, 1215-1223, doi:10.1021/np500088u (2014).

2 Pham, C. D. *et al.* Aaptamine Derivatives from the Indonesian Sponge Aaptos suberitoides. *J Nat Prod* **76**, 103-106, doi:10.1021/np300794b (2013).

3 Yu, H. B. *et al.* Cytotoxic Aaptamine Derivatives from the South China Sea Sponge Aaptos aaptos. *J Nat Prod* **77**, 2124-2129, doi:10.1021/np.500583z (2014).

4 Chen, M. H. *et al.* Alkaloids from the Root of Isatis indigotica. *J Nat Prod* **75**, 1167-1176, doi:10.1021/np3002833 (2012).

5 Tang, Y. *et al.* Casuarinines A-J, Lycodine-Type Alkaloids from Lycopodiastrum casuarinoides. *J Nat Prod* **76**, 1475-1484, doi:10.1021/np4003355 (2013).

6 Song, W. *et al.* Uralsaponins M-Y, Antiviral Triterpenoid Saponins from the Roots of Glycyrrhiza uralensis. *J Nat Prod* **77**, 1632-1643 (2014).

7 Ibrahim, M. A. *et al.* Minor Diterpene Glycosides from the Leaves of Stevia rebaudiana. *J Nat Prod* **77**, 1231-1235 (2014).

8 Campana, P. R. V., Coleman, C. M., Teixeira, M. M., Ferreira, D. & Braga, F. C. TNF-alpha Inhibition Elicited by Mansoins A and B, Heterotrimeric Flavonoids Isolated from Mansoa hirsuta. *J Nat Prod* **77**, 824-830 (2014).

9 Cheng, Y. B. *et al.* Limonoids from the Seeds of Swietenia macrophylla with Inhibitory Activity against Dengue Virus 2. *J Nat Prod* **77**, 2367-2374 (2014).

**Visualization of 10D embeddings using 10 randomly selected compound families and 3 randomly selected examples from each family (diagonal is the projection for every compound on that axis)**


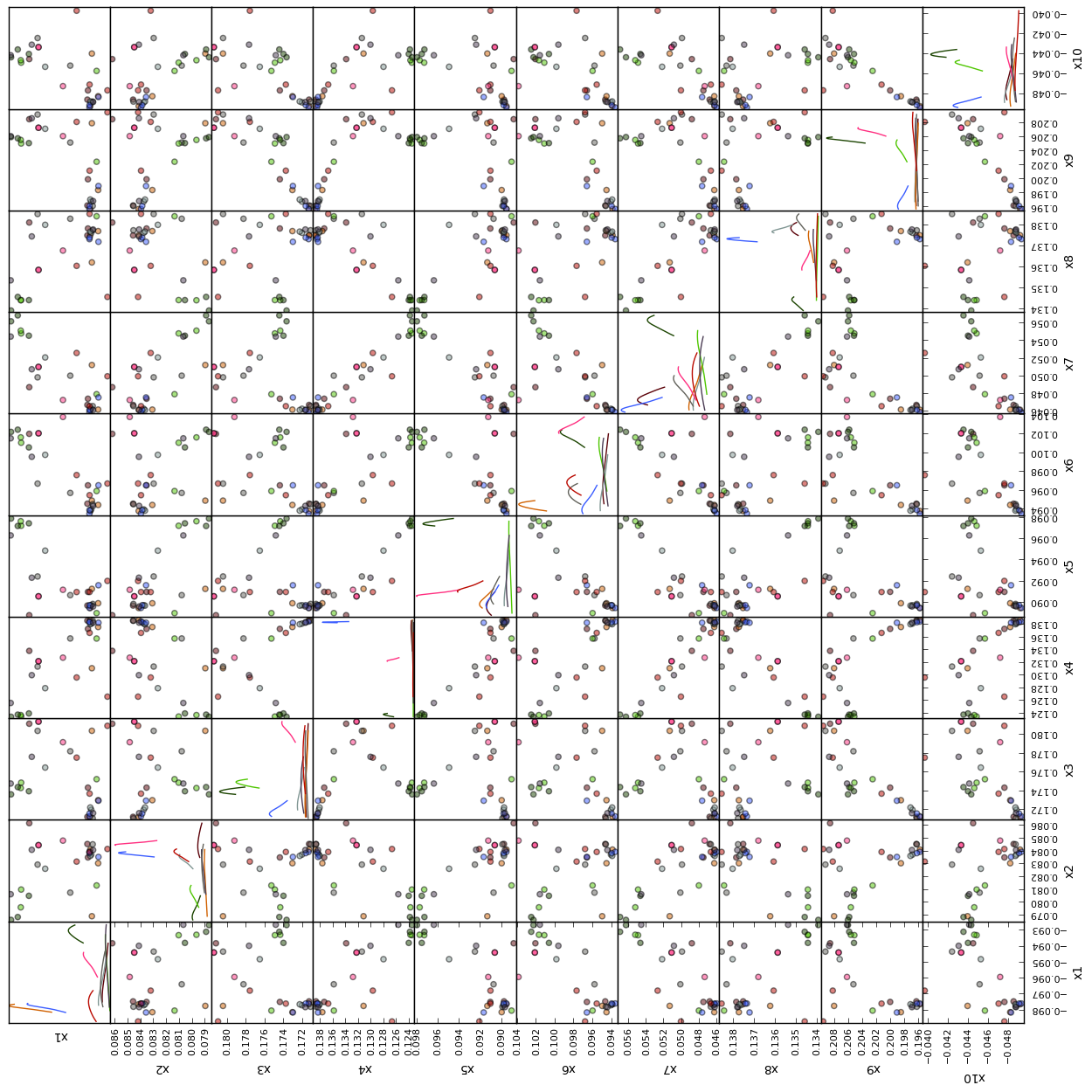


**Total number of pairs of inputs in the training process**

A siamese network is comprised of a pair of identical networks that are trained with pairs of inputs. For SMART5: there are 5,982 positive pairs, and 2,103,476 negative pairs. For SMART10 there were 3,787 positive pairs and 410,718 negative pairs. Notice that number of pairs grows with an order of O(n^2). During training, the positive and negative pairs must be balanced. We generated minibatches of 200 with 100 pairs randomly chosen from the positive pair set, and 100 from the negative pair set. The pairs are resampled each time.

This training strategy explains one reason why a siamese network is appropriate for this task. To train a deep network, typically on the order of a million examples are used. The advantage of the siamese network is that they amplify a small data set by training on pairs as opposed to single examples. A second reason why siamese networks are appropriate is that we need a cluster space, rather than a classification of compounds into families. If the system were simply trained to take an example and classify it, this would not be appropriate for new compounds for which the category is unknown. The cluster map generated by SMART places the new compounds into a similarity space with known compounds.

**Reasons for software and parameters selection**

There exist a number of different frameworks to perform deep learning, including Torch, Tensorflow, Theano, Caffe, mxnet, etc. There is no particular advantage of one over another, except for the tradeoff between ease of use and flexibility. The authors of this manuscript were most familiar with Theano, and at the time of project's start, this was the version of a deep learning framework that had a good (native) python interface, as well as a stable development version.

The choice of parameters and hyperparameters is completely empirical in the entire deep learning field, with no best method except trial and error. The reported parameters led to good results in all experiments. When searching for hyperparameters, we looked into GPU utilization/runtime per iteration. There is a tradeoff between batch size, number of iterations of learning, and wall-clock time. GPUs are very good at processing batches of examples for training, and the more memory a GPU has, the larger batch it can process (higher utilization), which will reduce the noise in Stochastic gradient descent (SGD). However, the larger the batch, the more time it requires, but this in turn, reduces the number of SGD iterations that are required. Another essential parameter to tune is the learning rate; if the learning rate is too high, the optimization procedure can diverge, whereas smaller rates may terminate before reaching the best minimum of the objective function. Based on preliminary experiments, we chose a batch size of 200 pairs and an initial learning rate of 0.002. Other parameters were likewise chosen based on preliminary experiments, for example: margin = 0.02, l2 regularization multiplier on dense layers: 0.0001, update rule = adagrad.

**SMART training speed**

Our initial experiments on SMART5 with k=2 dimensional embedding were run for 100k iterations on an Amazon EC2 g2.2xlarge instance (using NVIDIA GRID K520 GPU) which required 8 days. Using batch normalization, the time was reduced to 28 hours. Our final experiments on SMART5 were run on an Nvidia Titan X (Maxwell), and we limited the number of iterations to 15k; this was completed in 3-4 hours.

**Noisy HSQC spectra of ebractenoid C**

**
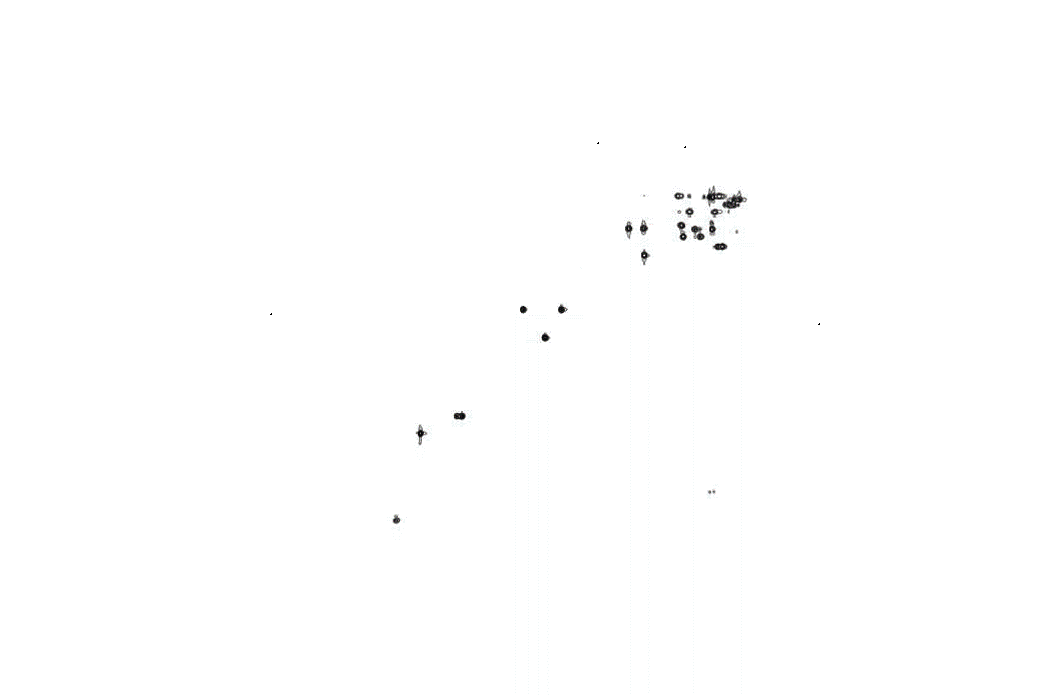

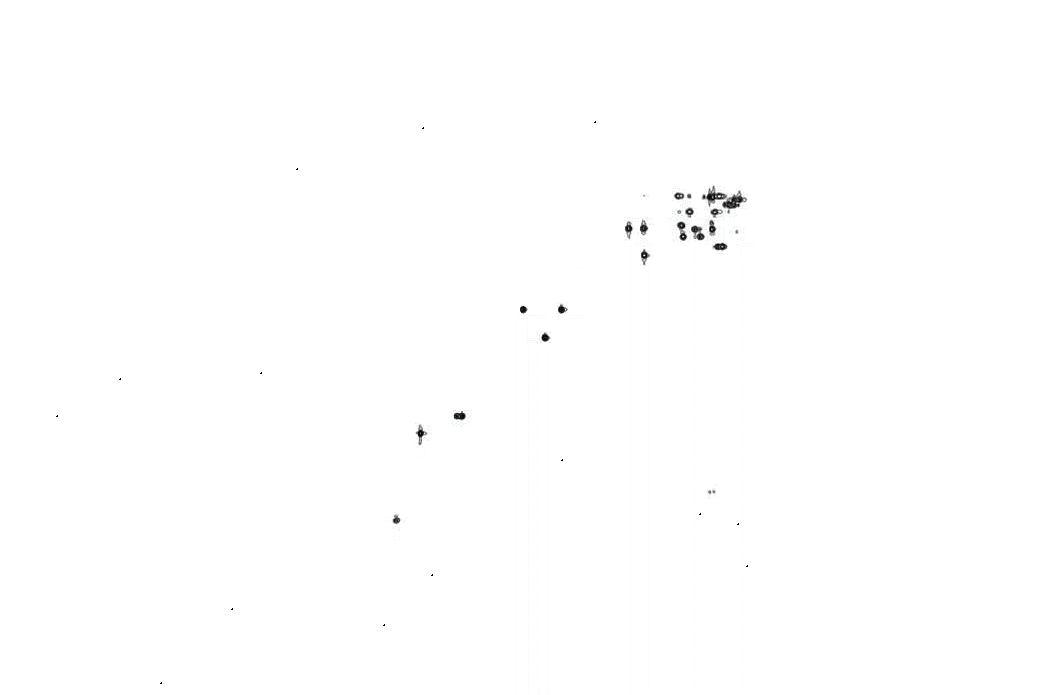

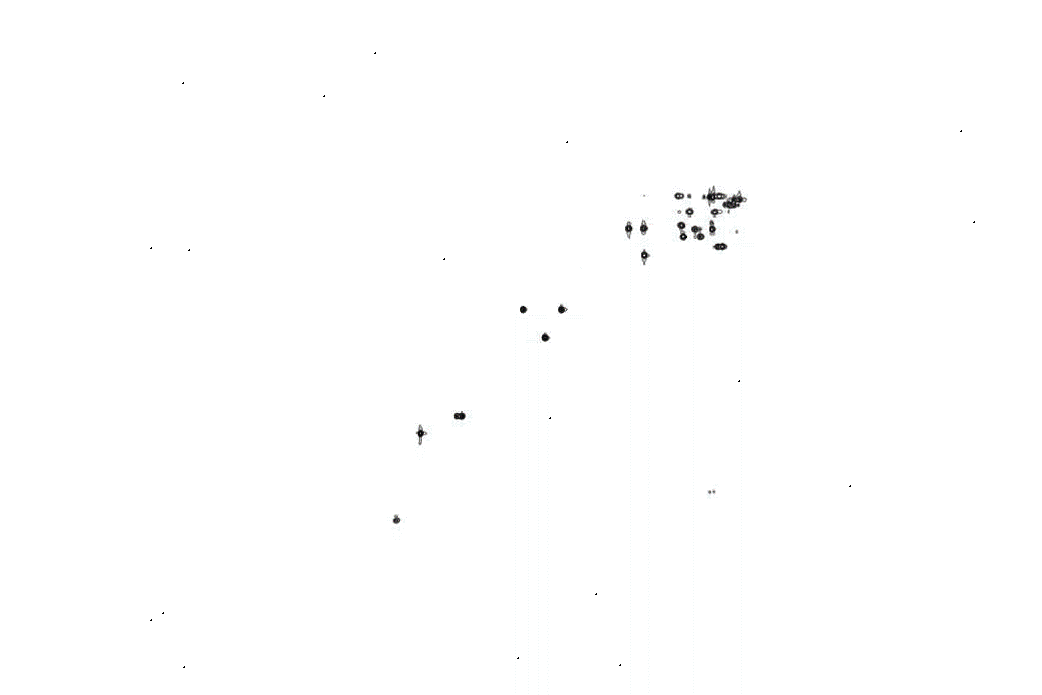

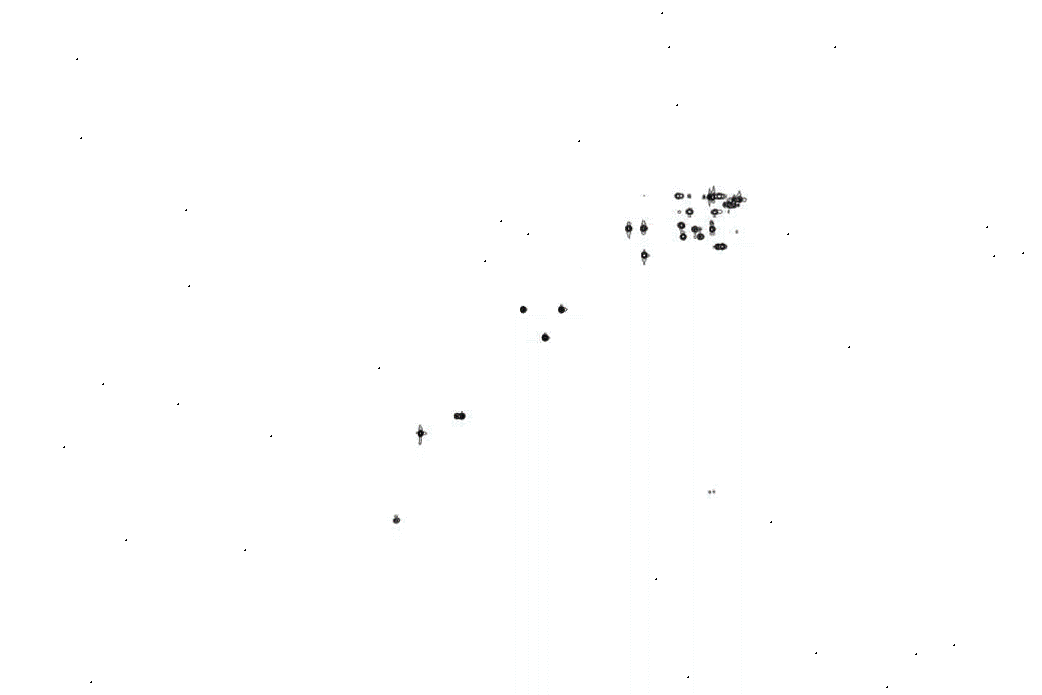
**

**
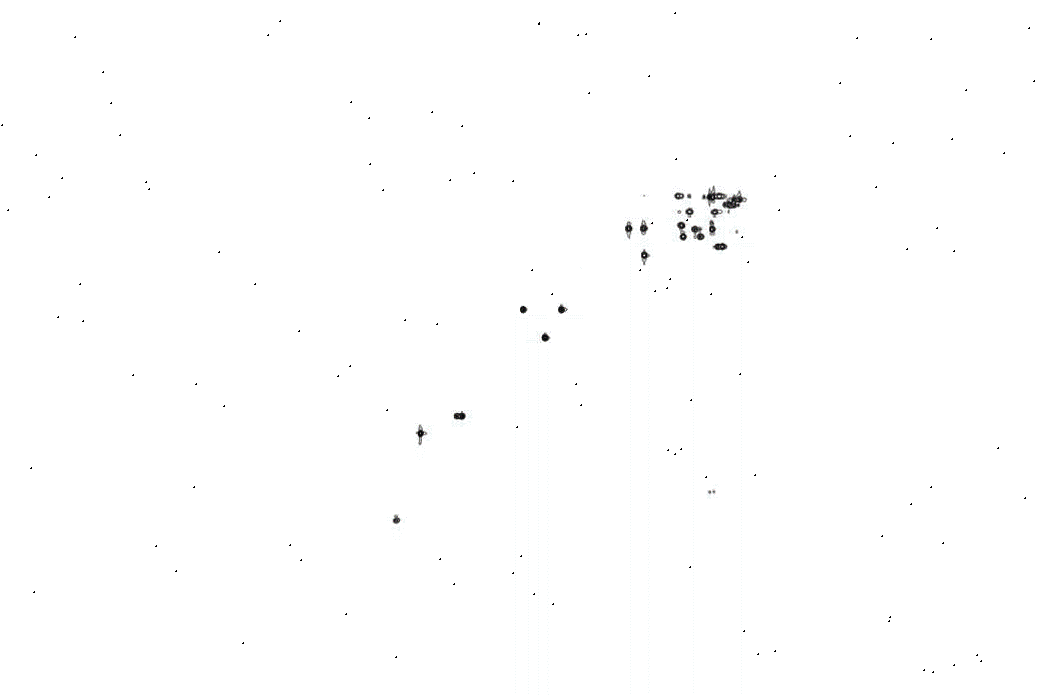

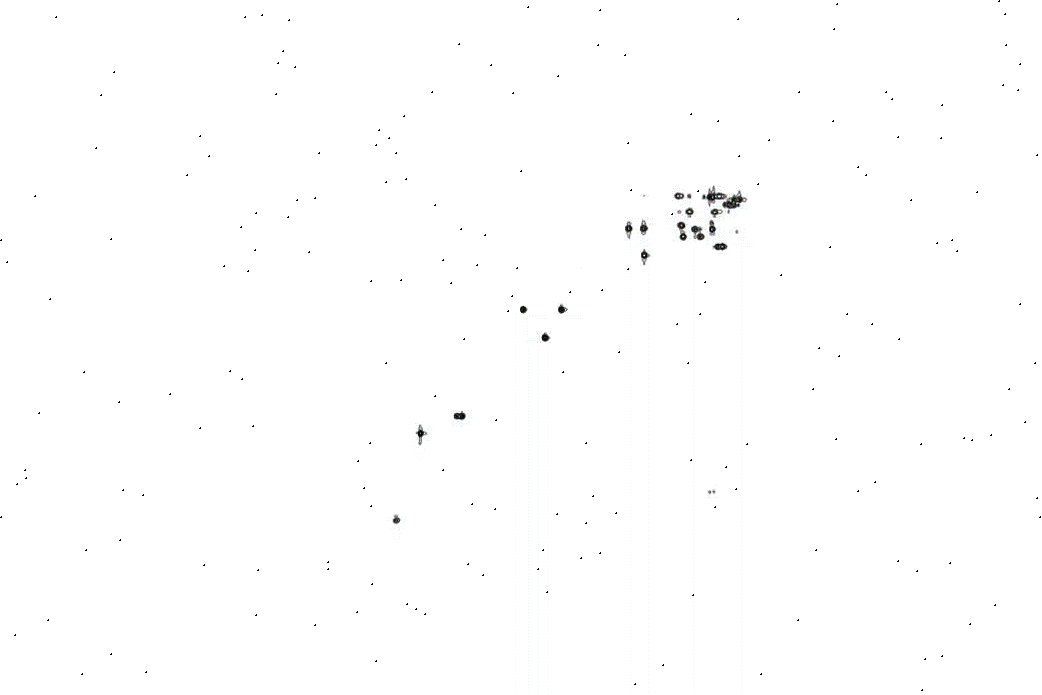

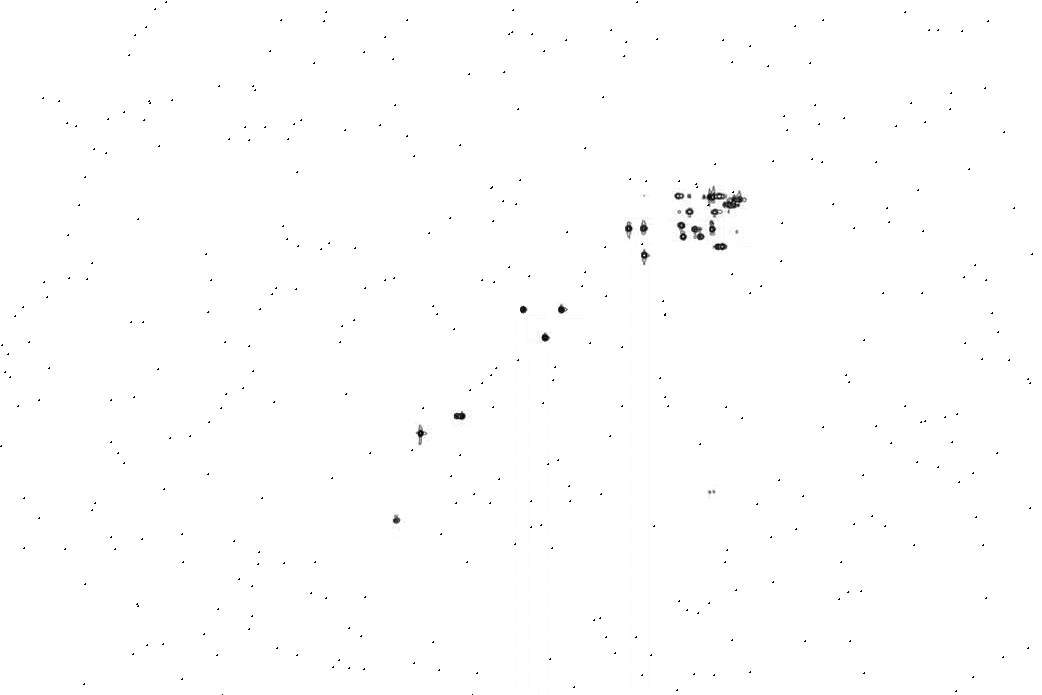

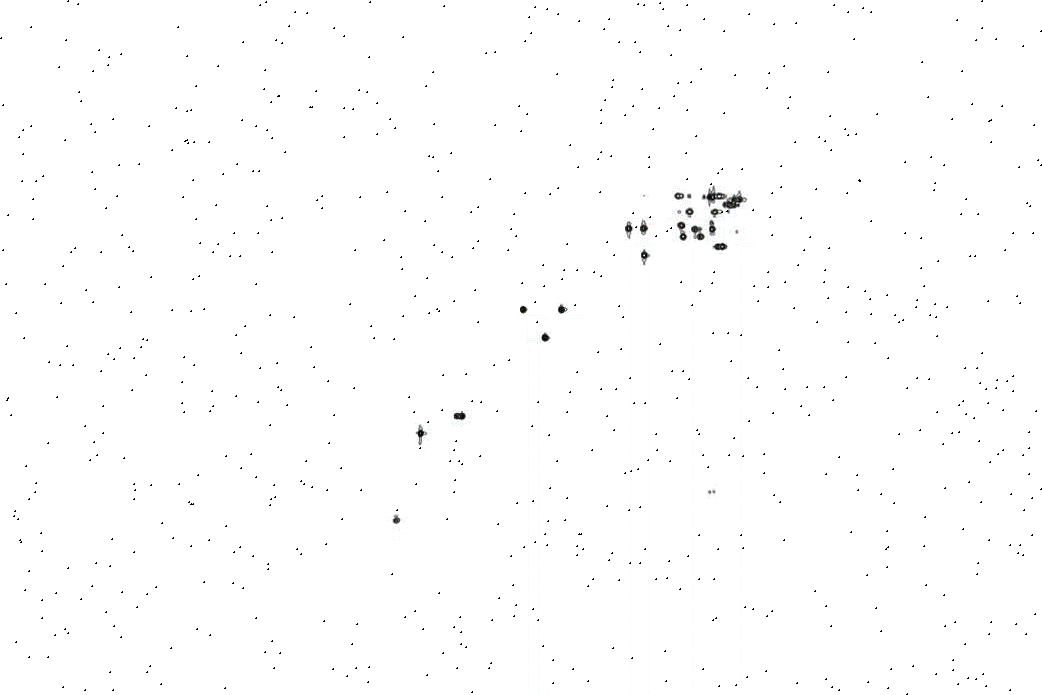

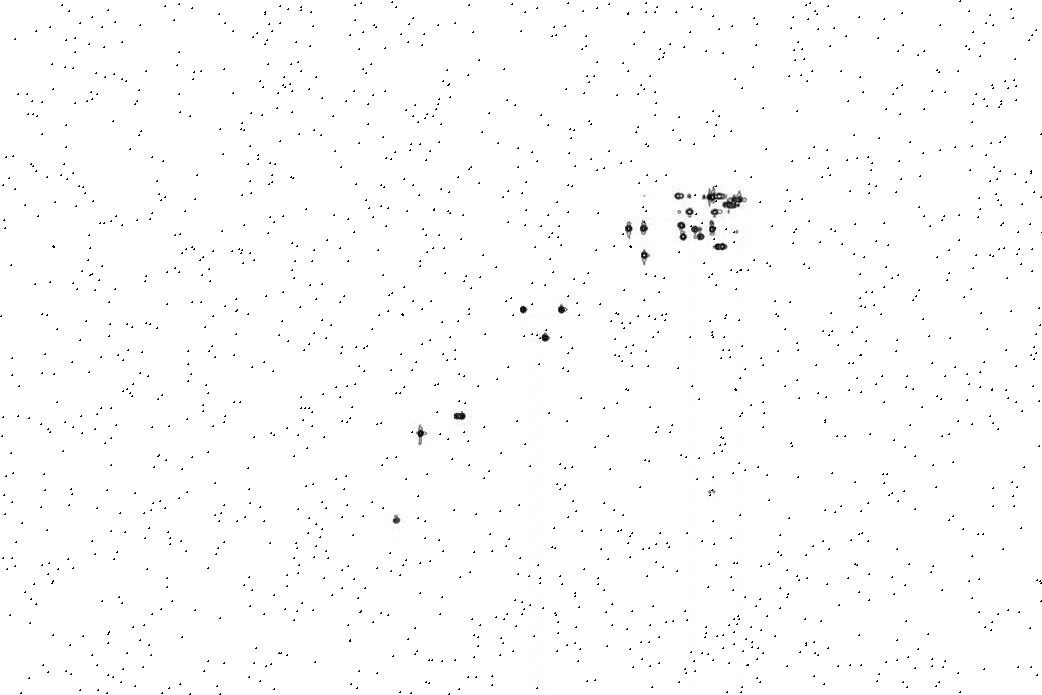

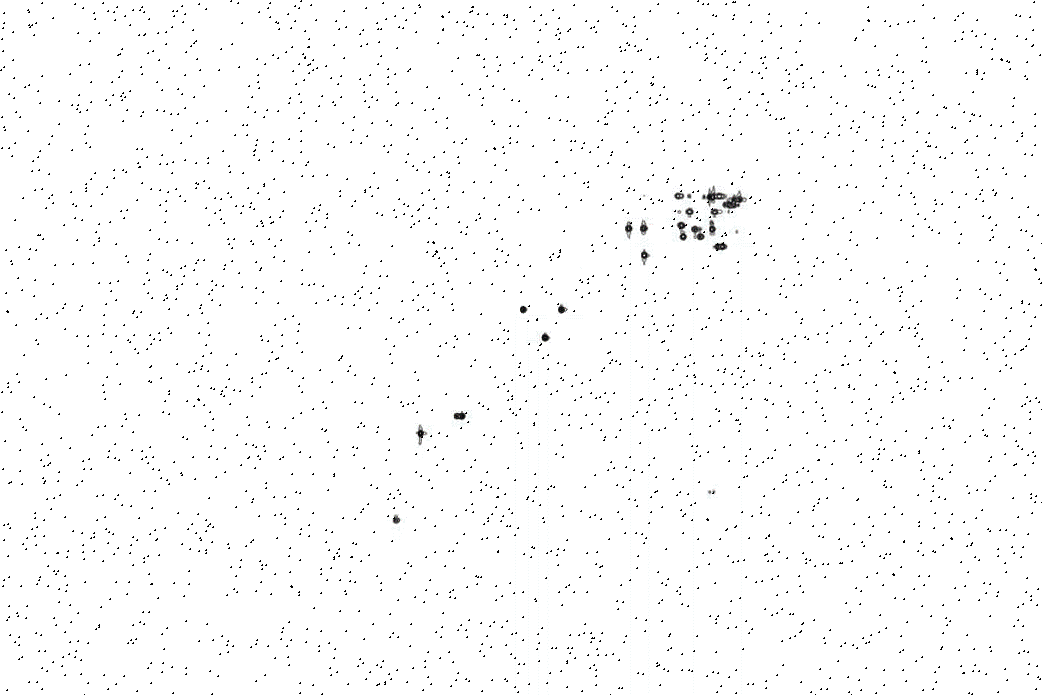

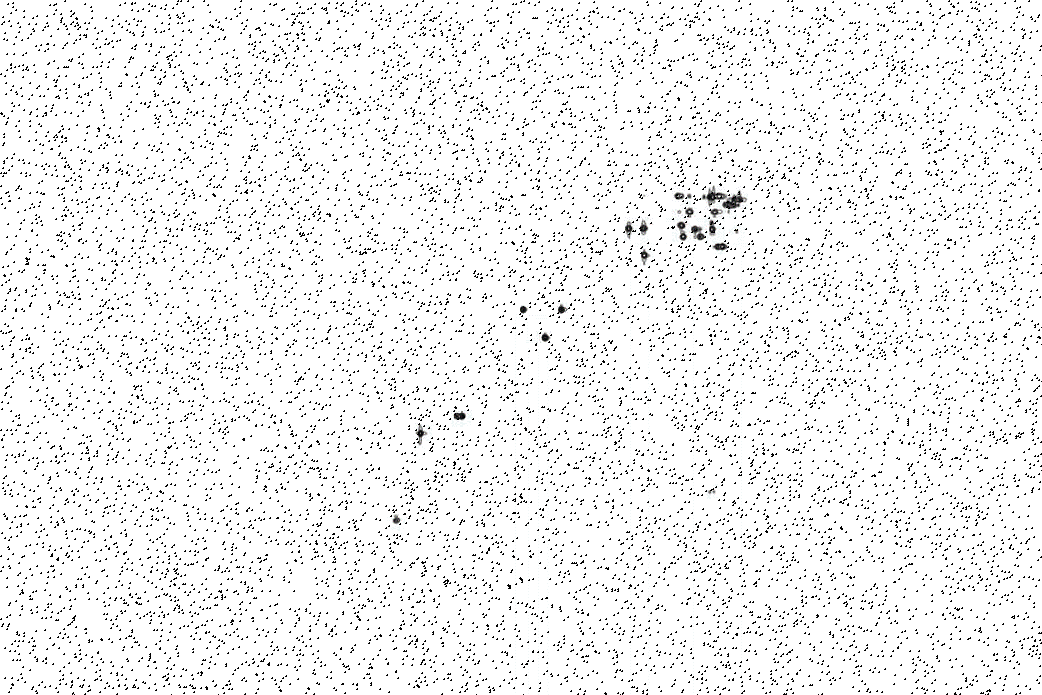

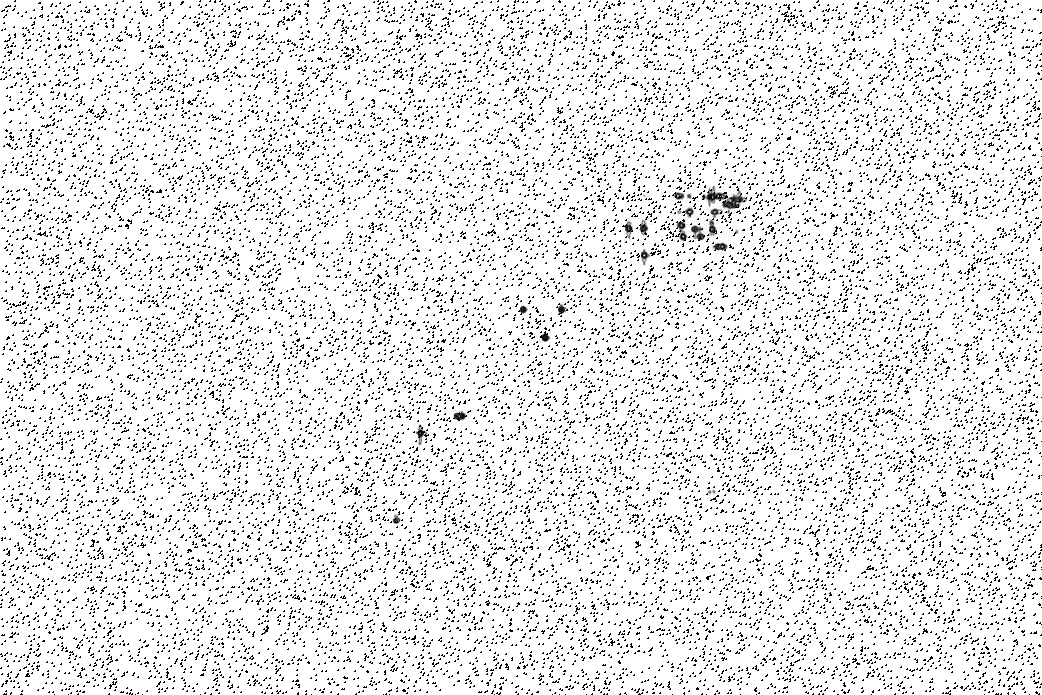
**

**Noisy HSQC spectra of hyphenrone I**

**
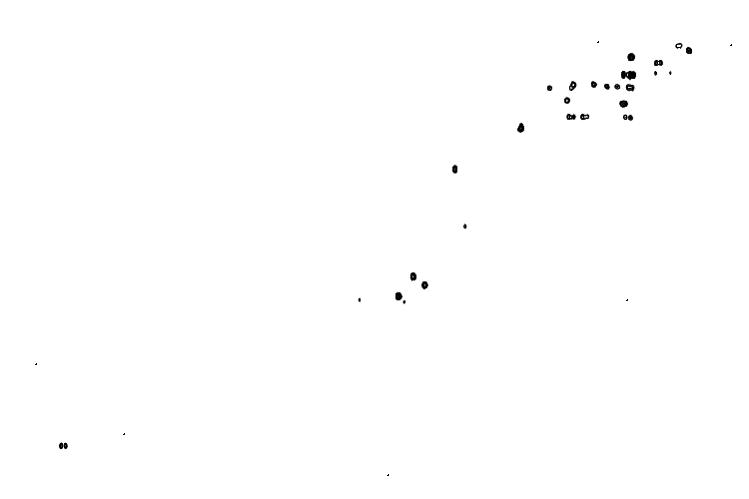

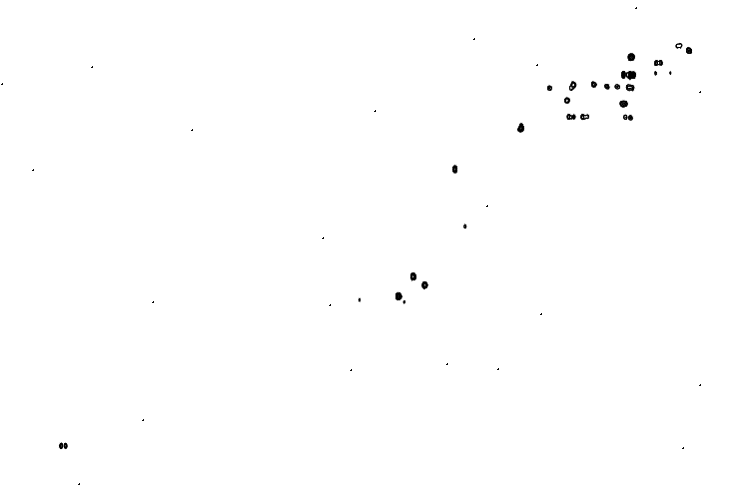

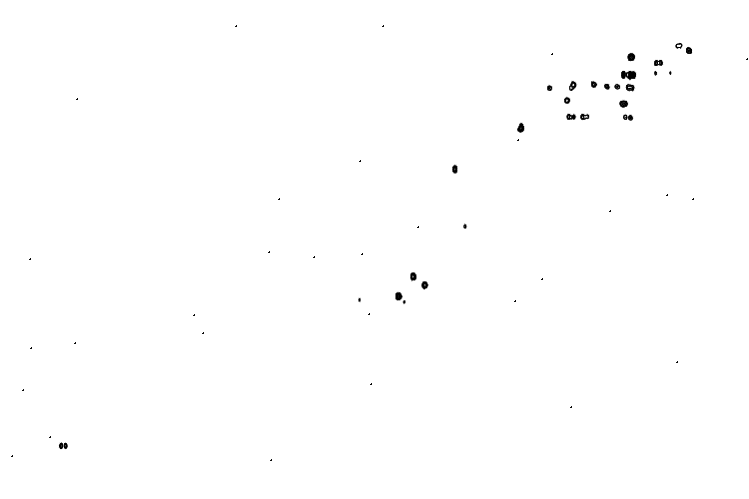

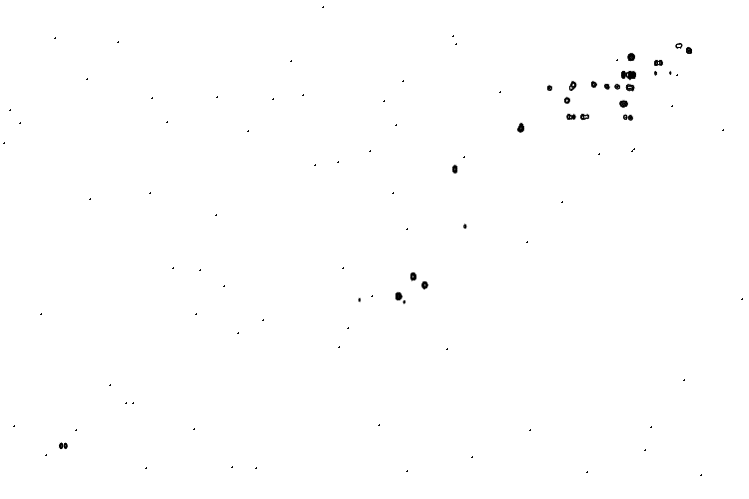

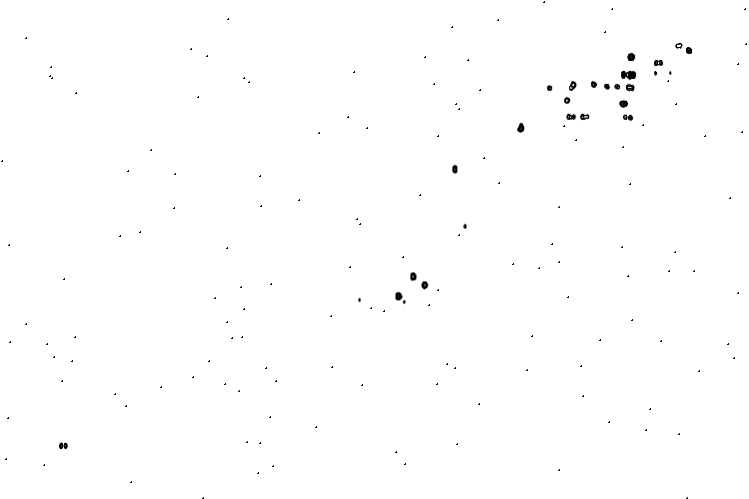

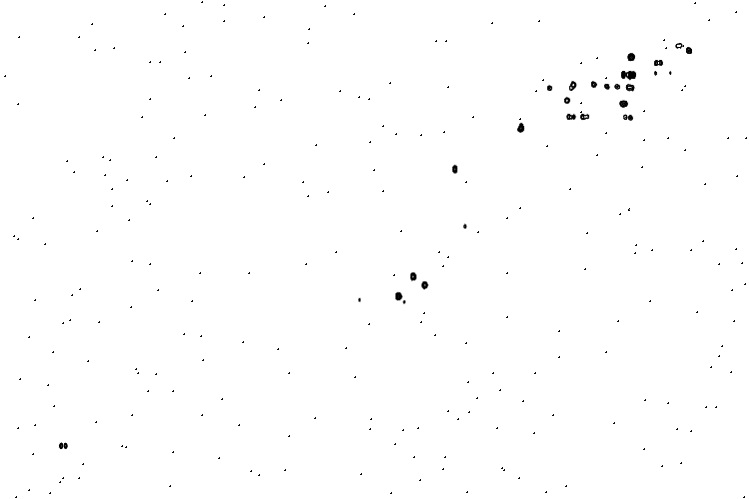

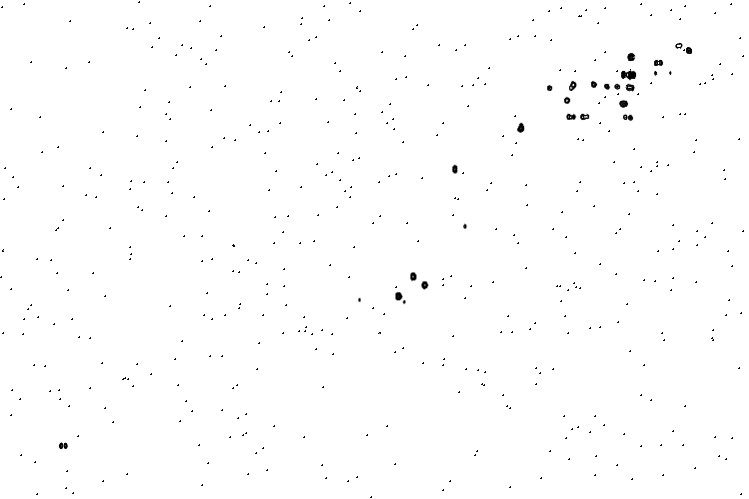

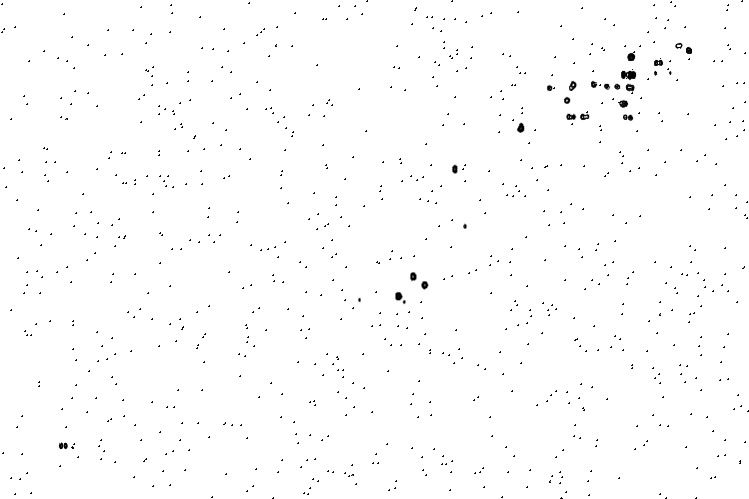

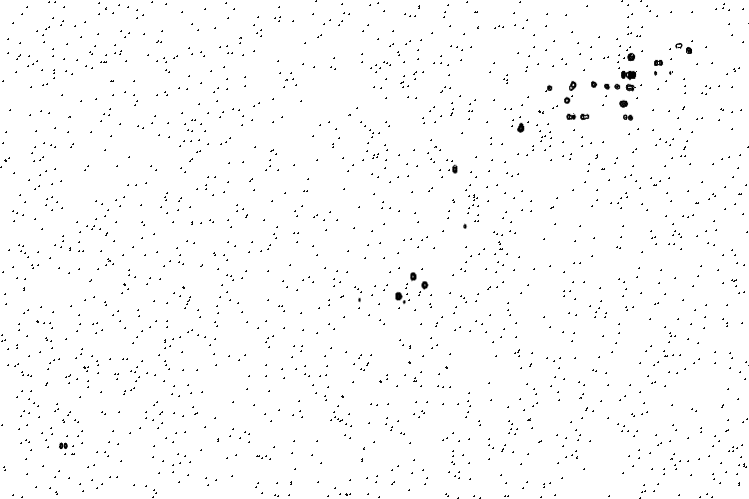

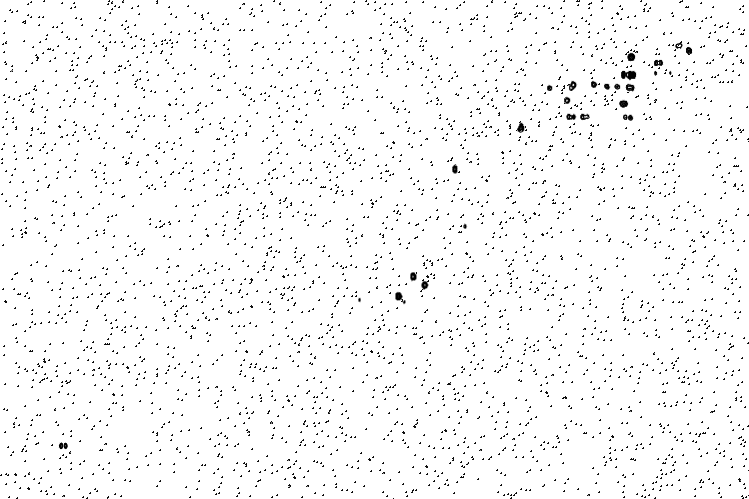
**
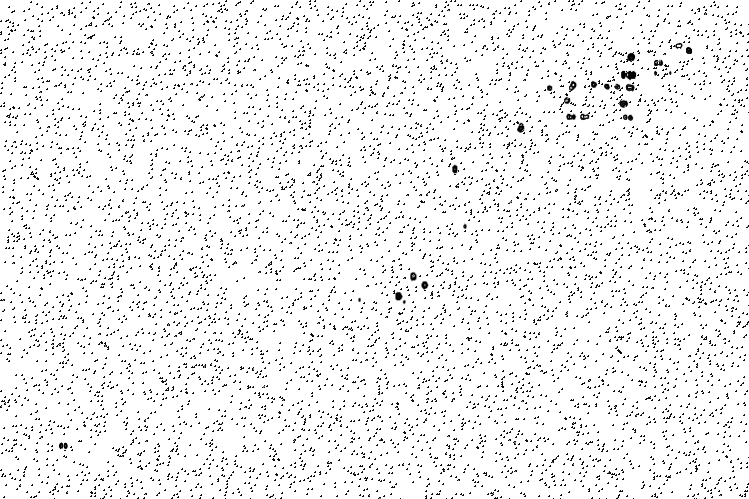

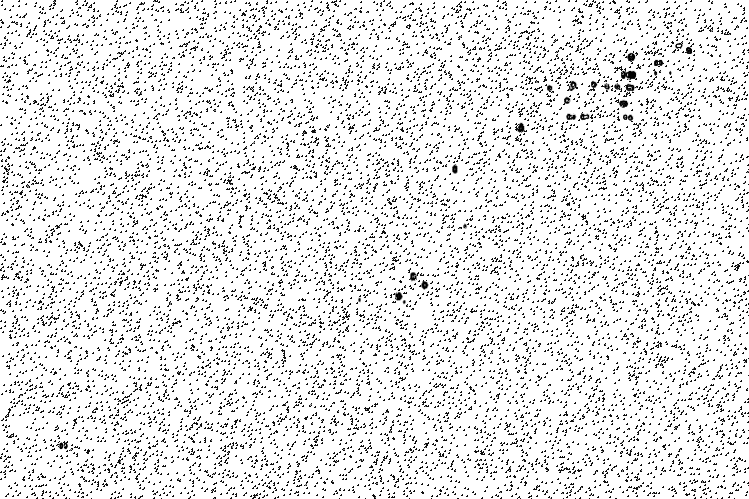

Supplement: Supplementary file 1 — Supplementary Information [file 41598_2017_13923_MOESM1_ESM.doc]
